# Criticality assessment of metal resources in China

**DOI:** 10.1016/j.isci.2021.102524

**Published:** 2021-05-08

**Authors:** Wenyi Yan, Zhaolong Wang, Hongbin Cao, Yi Zhang, Zhi Sun

**Affiliations:** 1 Beijing Engineering Research Center of Process Pollution Control, Division of Environment Technology and Engineering, National Engineering Laboratory for Hydrometallurgical Cleaner Production Technology, Institute of Process Engineering, Chinese Academy of Sciences, No. 1 Beierjie, Zhongguancun, Haidian District, Beijing 100190, China; 2 University of Chinese Academy of Sciences, Beijing 100190, China; 3 Solid Waste and Chemicals Management Centre, Ministry of Ecology and Environment, Beijing 100029, China

**Keywords:** environmental science, materials science, materials class, metals

## Abstract

With the development of modern industries, the sustainability of critical resources has attracted worldwide attention considering the entire supply chain. With a large industrial sector size in China, a safe supply of metal resources is crucial to ensure the effective operation of the whole industry. Although specific criticality analyses have been applied to identify critical resources in some regions, including Europe and the USA, they are not ready to be directly applied in the case of China because the structure of China’s industry is remarkably different from other areas. In this research, a three-dimensional methodology considering supply safety, domestic economy, and environmental risk is demonstrated, where Chinese industrial conditions are specifically considered. In total, 64 materials were introduced to perform the criticality assessment, and 18 metals were classified with a high criticality degree in the three-dimensional criticality space. With the obtained findings decision-makers can formulate strategic deployment to promote resource management.

## Highlights

• A systematic evaluation methodology for metal resource criticality was established
• Effects of supply risk, economic change, and environmental risk were considered
• 64 materials were introduced to perform the criticality assessment
• 18 metals were classified with a high criticality degree in China

## Graphical abstract

**Figure:**
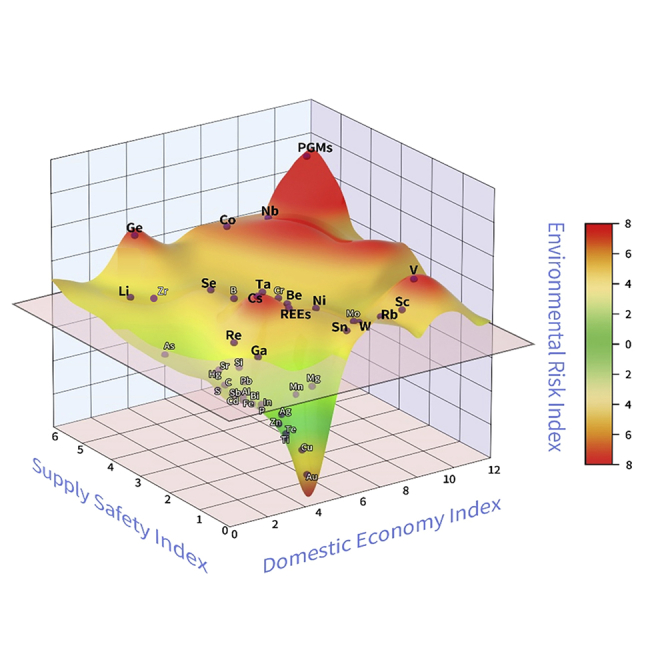

## Introduction

Metal resources, which are foundational to economic and social development, are crucial for ensuring the sustainable development of the global manufacturing industry. In the 21st century, the consumption of critical metals increased sharply, leading to a greater demand and supply risk of global metal resources (Eggert and Roderick, 2011; Hayes and McCullough, 2018). To cope with the potential supply risk of mineral resources in the future, many countries have taken effective measures (China, Ministry of Land and Resources of the People's Republic of China, 2016a; Gardner and Colwill, 2018; Rabe et al., 2017). To minimize imports and dependence, the United States is accelerating the security of supply by actively promoting the extraction and recycling of domestic critical mineral resources (DOE. U.S. Department of Energy, 2019; US Department of the Interior, 2018). The European Union (EU) strengthened mineral resource development within the region and promoted safe access to raw materials to secure a stable supply of critical minerals (Bach et al., 2017; Claudiu et al., 2016; SETIS. Strategic Energy Technologies Information System, European Commission, 2015). Moreover, Canada and Australia have improved the quality and efficiency of mining development. Accordingly, the Chinese National Plan for Mineral Resources (2016-2020) (China, the State Council of the People's Republic of China, 2016c) clearly pointed out that it was necessary to further implement the national security strategy and improve management to guarantee the safe supply of resources. In the future, ‘'resource competition’ is predicted to become increasingly fierce worldwide to ensure resource security (Bram and Henrike, 2011).

At present, 162 types of mineral resources have been found in China (China, Ministry of Natural Resources of the People's Republic of China, 2020). As a country with the largest mineral processing industry, China has a complete processing and utilization system involving exploration, mining, refining, and synthesis. The mining industry has become an important pillar of China’s national economy, and its contribution accounts for more than 30% of the gross domestic product (Ju et al., 2019). China has established cooperative relations with more than 100 countries and regions in the exploration and mining of energy resources in recent years, which plays a significant role in promoting the development of global industry. Therefore, the strategy of metal mineral resource management in China has a significant impact on the global mineral market.

Unlike other developed countries, the characteristic of the Chinese manufacturing industry is that most are resource-intensive and labor-intensive and focus on front-end and low-end processes. The economic structure characterized by heavy industrialization and extensive development models has become the main incentive for green development in Chinese industries (China, the State Council of the People's Republic of China, 2016b). In this case, sustainable development strategies promulgated by other countries are not applied to China owing to their uniqueness. It is necessary to conduct an overall assessment of Chinese metal resources and environmental status in order to provide a reference for policymakers to carry out top-level design for a sound industrial system.

A good understanding of current or potential future situations of criticality of resources can help stakeholders make better decisions to mitigate criticality issues or take measures in advance. Governments and researchers worldwide have attached great importance to the evaluation of mineral resource criticality, and several assessment methodologies have been established (Schrijvers et al., 2020; Blengini et al., 2017; European Commission, 2010; Graedel et al., 2012; Fu et al., 2018). In recent years, the United States (DOE. U.S. Department of Energy, 2010; DOE.U.S. Department of Energy, 2011), the EU (European Commission, 2010, 2014, 2017, 2020), and other developed countries (Hatayama and Tahara, 2015; Marscheider-Weidemann et al., 2016) have issued critical material lists and considered in the national development strategy. In 2010, the EU Raw Materials Supply Group listed 14 critical raw materials (European Commission, 2010) by considering the impacts of supply risk and economic importance. They were updated in 2014 (European Commission, 2014), 2017 (European Commission, 2017), and 2020 (European Commission, 2020), respectively, and 30 types of materials have been identified as critical in the latest version. In 2012, the US Department of Energy issued a critical material strategy (DOE. U.S. Department of Energy, 2011). From the perspective of energy metals demand in the US, 14 types of materials have been categorized as critical. The evaluation indicators were divided into two groups: the materials’ contribution to clean energy and supply risk. The list was upgraded in 2018, in which 35 raw materials with high dependence on imports and economic importance were identified as critical (Fortier et al., 2018). The study published by Ioannidou et al. (2019) suggested the incorporation of dynamic parameters that reflect changes over time to enhance the criticality assessment framework. Helbig et al., (2021) illustrated the basics of criticality assessments by the examples of copper and indium and summarized four steps to conduct a criticality assessment. These two reviews (Ioannidou et al., 2019; Helbig et al., 2021) were comprehensive and brought us inspiration and suggestions for improving evaluation methods. In China, the criticality of several metals (Eheliyagoda et al., 2020) and the supply risk used in lithium-ion batteries (LIBs) (Song et al., 2019; Yan et al., 2020) have been evaluated. There are four types of critical materials (lithium, cobalt, nickel, and graphite) in LIBs, and priority should be given to the recycling of lithium and cobalt specifically. A list of strategic minerals for China was issued in 2016, and its supply safety has been analyzed by considering import reliance and reserves concentration (China, the State Council of the People's Republic of China, 2016c; Wang et al., 2019), which could not be equated with critical materials with different evaluation criteria.

However, there is still no unified understanding of the connotations of critical mineral resources in China. In fact, the list of critical materials should be evaluated in a particular scope, as it varies with time and location owing to the differences in data sources and evaluation dimensions. As mentioned above, the dimensions set in the current evaluation methods consider the influence of supply risk and economic fluctuation with different sub-indicators and weight combination, which is inadequate and incomplete for the Chinese context. Furthermore, their evaluation target did not fully accommodate Chinese resource strategy. There is an urgent need for a new integrated investigation on critical metals assessment in China, but detailed and comprehensive research focusing on criticality is limited.

In this study, an integrated evaluation methodology for metal criticality is established based on the consideration of the entire life cycle from mining and refining to recycling. The evaluation is quantitative, and the latest data are adopted to improve transparency and accuracy. Individual metals are located in a three-dimensional ‘criticality space’ with the axes of supply safety, domestic economy, and environmental risk indices. It is noteworthy that the characteristics of material value endowed by the market are introduced in this methodology, as the real-time economic market is the feedback and wind vane of global metal resources and related industries. In addition, the environmental risk dimension is considered according to the Chinese special economic structure and environmental status quo. In detail, evaluations of each dimension involve a number of criticality-related indicators: Supply safety considers the impacts of net import reliance, substitutability, secondary resources (recycling rate), policy potential index, and concentration of resource distribution; the domestic economy index involves the influences of end-use value and market value of metals; toxicity risk and pollutants released in the production process of metals are incorporated in the environmental risk assessment. This methodology was applied to the criticality assessment of 64 elements. A list of critical metals for China is obtained, which will be helpful for Chinese decision-makers and enterprises to actively respond to the international mining situation, alleviate the potential risk of resource supply, and lay out the emerging strategic technology industries.

### Methodology

The purpose of this study was to evaluate the criticality of metal resources in China. A comprehensive and thorough assessment methodology was established, including three dimensions: supply safety, domestic economy, and environmental risk. In this context, supply security is an important influential dimension of sustainability. The dimensions of supply risk and economic importance are derived from the approach published by the EU raw materials supply group (European Commission, 2010). However, in contrast to the EU method, the indicators in our method were reintegrated and optimized. In particular, the criticality of resources is also closely related to market economic value, which has been ignored in previous studies. For example, after the implementation of relevant policies to promote the development of the global new energy vehicle industry, the prices of lithium and cobalt have increased significantly; it is stipulated in the new Chinese national standard that vanadium alloying elements must be added to rebar and the price of vanadium has risen by 100%, which has attracted significant attention in the market. Hence, the parameter indicating the characteristics of the material value endowed by the market is introduced. In addition, the future development layout of a country should consider both economic construction and sustainable development of the ecological environment. China’s economic industry is mainly a heavy chemical industry with extensive development, which has caused a lot of waste of resources and serious environmental pollution. In this context, the environmental risk dimension involving the impacts of toxicity of metal resources and waste emissions to the environment should be evaluated to provide information for policymakers to promote the construction of an ecological civilization.

With these improvements, this methodology is applied to the criticality assessment of 64 resources; when the value of each dimension increases, the criticality will increase. The selection basis and consideration of the three dimensions are shown in detail in the Supplementary File A. The scope of this study comprises the following.

#### Scope

##### Goal

The objective of this work is to develop a comprehensive methodology for assessing criticality and applying the concept to raw materials selection. These assessments will help governments and enterprises take appropriate measures to mitigate supply restrictions, such as focusing more on critical metals in the economy and policy, actively promoting the research and development of extraction, recycling, and alternative materials of critical metals.

##### Geographical coverage

This is the first time that the criticality of metal resources was investigated in China. However, as noted previously, criticality assessments have been carried out at the level of several developed countries in recent years, adopting diverse criteria and various time ranges. Inconsistent results were generated in terms of different data sources and aggregation forms of the criticality methods. From this perspective, the classification of critical materials will be significantly different with geographical scope. In this study, the geographical scope was within China.

##### Evaluated materials

**Figure 1:**
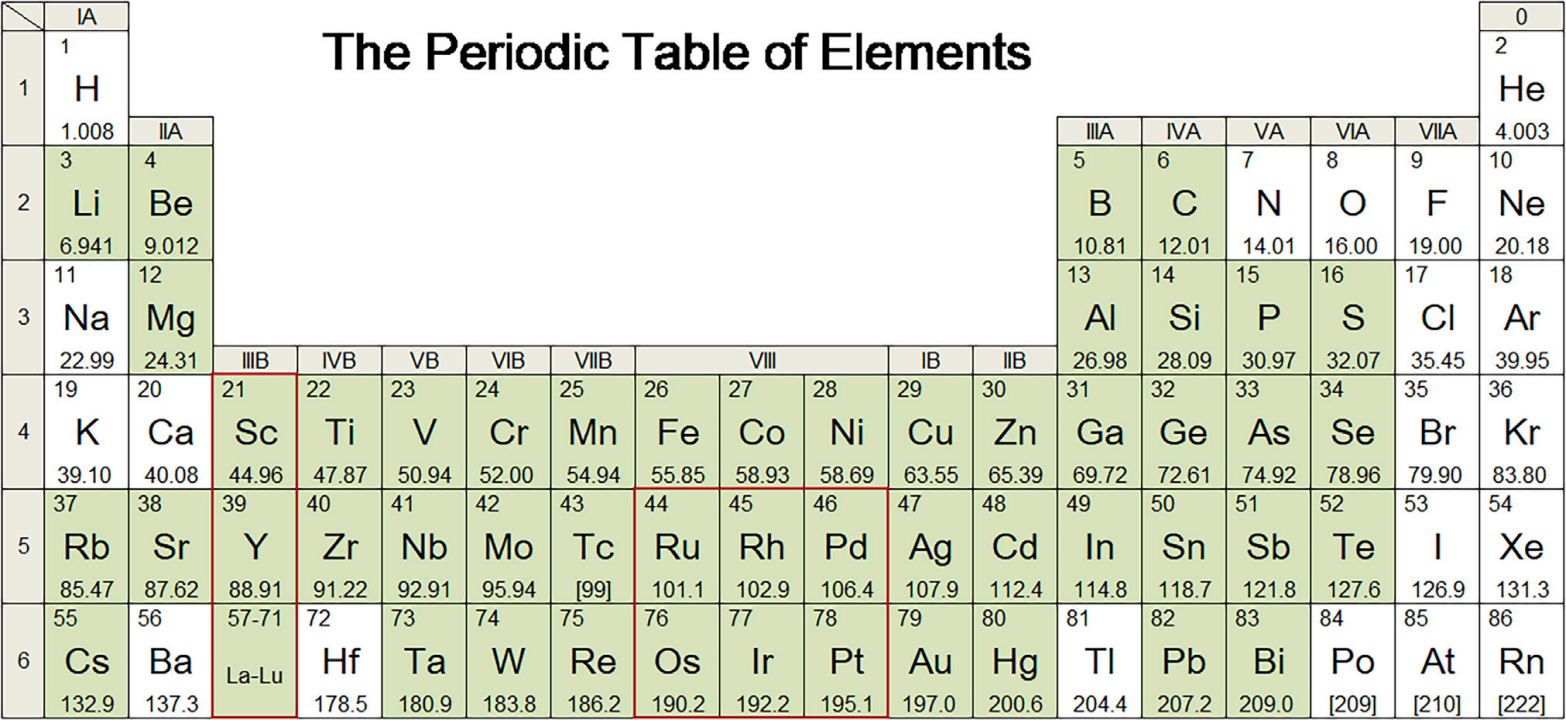
Distribution of evaluated elements in the periodic table of elements

The evaluation materials in this study emphasize metals and non-energy minerals, which cover 64 elements in the periodic table of elements except actinides, gas elements, and materials with relevant data unavailable and minimal demand (Figure 1). It is necessary to assess the criticality of these materials because of their indispensable roles in human life and industrial production. The name of the metal was used to indicate the situation of its corresponding ore.

##### Time horizon

Criticality evaluation captures the relative criticality of materials at a specific time point, so the results are not fixed. The criticality of raw materials for the next five years was evaluated and analyzed in this study. Priority is given to the latest public statistical data. Data from the past five years (2015-2019) were generally used. Exceptions are explained in the context of specific circumstances. To provide a long-term list of critical materials, it is necessary to address the changing situation and update the data regularly.

#### Approach

**Figure 2:**
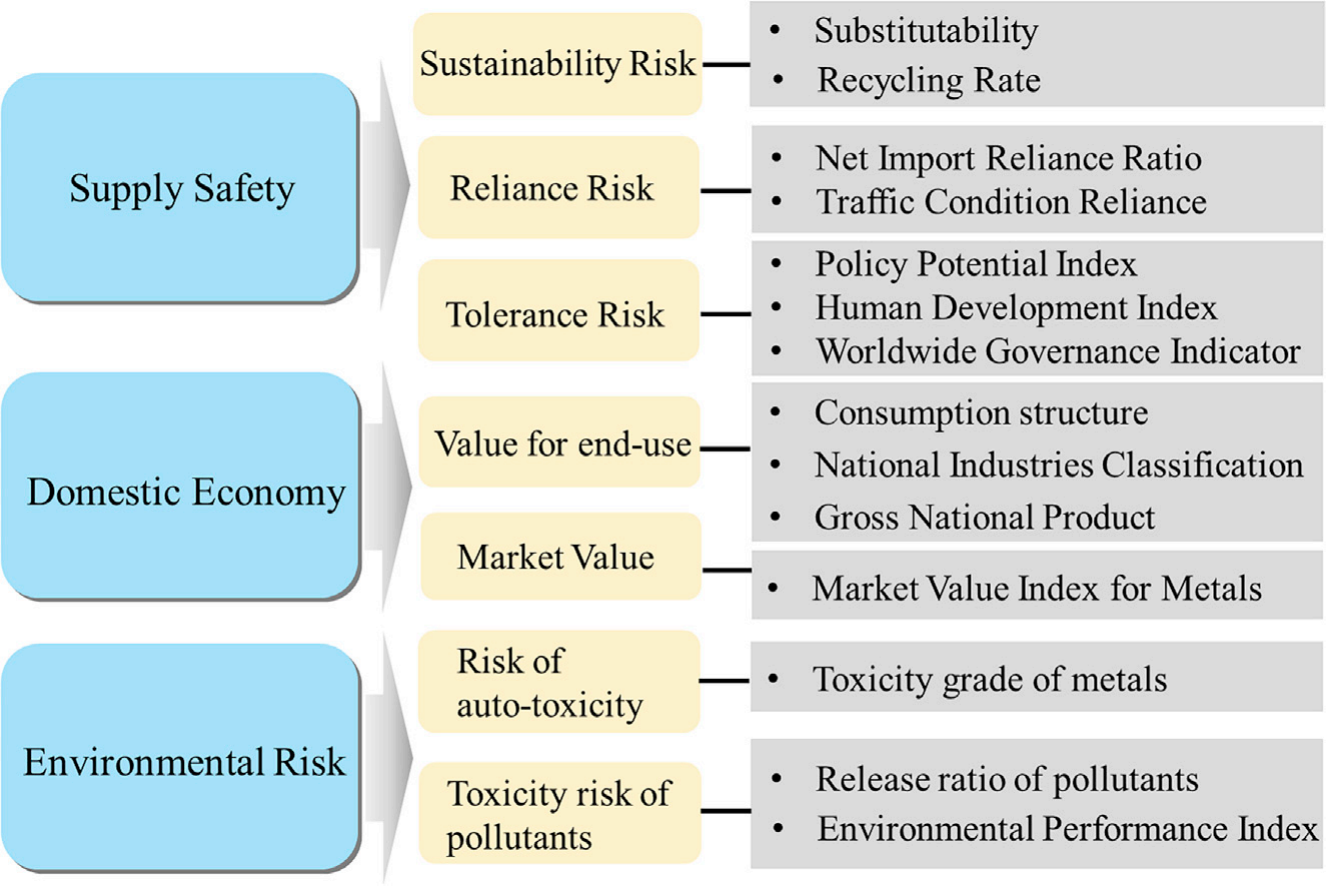
Schematic of the criticality assessment methodology

When the supply risk of a material and its impact on the economy and environment are higher than those of most other materials, the material is considered critical. A three-dimensional system was designed, including the impacts of supply risk, economic fluctuation, and environmental risk. Figure 2 shows a schematic of the criticality assessment methodology. The details of these three dimensions are elucidated as follows.

##### Supply safety index (*SS*)

The supply safety index aims to characterize the supply risk of materials. It includes three aspects of risk: sustainability, reliance, and tolerance risk.

The potential for one material to be replaced by another in its application field affects *SS*. The substitutability index is introduced in this method, which is the aggregate of the substitutability of materials in each application field. The degree of material substitutability for each use is determined by experts in the industry (European Commission, 2014). If the substitutability value is 0, it means that it can be replaced at no cost; 0.3 indicates that it is feasible to be replaced at a lower cost; 0.7 means that it is feasible to be replaced with aer high cost; and 1 indicates that it is irreplaceable.

With the development of recycling technology, the supply of materials can come from not only primary resources but also secondary resources. If the recovery rate of a certain material is high, the contribution of secondary resources will be great, which can alleviate the supply risk in the future. The recovery rate of a certain material varies owing to the different waste products and technologies. Considering the reliability and unity of data sources, this study adopts the theoretical recovery rate of materials in published literature and reports (Graedel et al., 2011; USGS, 2020).

The dependence of material demand in foreign countries is also an important factor affecting *SS*. In this method, import reliance was introduced to indicate this effect, which is closely related to the production, consumption, import, and export of materials. The more we rely on imports, the greater the supply risk.

The Herfindahl-Hirschman index (HHI) is a comprehensive index to measure the degree of industrial concentration and has been widely used in anti-trust proceedings or assessments (European Commission, 2010). In this method, the HHI is used to evaluate the distribution concentration of metal resources. In the calculation of HHI, the economic and political stability of producing countries are also considered by involving the Worldwide Governance Indicator (WGI) published by the World Bank (WGI, 2019). If the value of HHI is high, the stability of the producing country will have a significant impact on the international market of the material. Emergencies in these countries will bring about supply risks.

The calculation formulas of supply safety index are described below:

(Equation 1) $$SS_{M}=SI_{M}TR_{M}\left(1-\rho_{M}\right)HHI_{WGI,M}$$

where *SS*_*M*_ represents the supply safety index of material M. *ρ*_*M*_ is the recycling rate of *M*. *SI*_*M*_ is the aggregate substitutability of *M*, and its calculation method is shown in Equation (2).

(Equation 2) $$SI_{M}=\sum_{1}^{\text{i}}SI_{M,\text{i}}S_{i}$$

where i represents the end use of material *M. S*_*i*_ refers to the proportion of *M* in end use I, and $\sum S_{i}=1$. *SI*_*M,i*_ is the substitutability of *M* in end use i.

(Equation 3) $$TR_{M}=\frac{Do+Im-Ex}{Do}$$

*TR*_*M*_ refers to the import reliance of material *M* and can be calculated using Equation (3). *Do* is the annual domestic production of material *M* in China. *Im* and *Ex* are the annual import and export volumes of material *M* in China, respectively.

(Equation 4) $$HHI_{WGI,M}=\sum {S_{M,j}}^{2}*WGI_{j}$$

The calculation formula for HHI is shown in Equation (4), where *S*_*M,j*_ is the production percentage of material *M* produced by country *j* in the total global production of *M*. *WGI*_*j*_ is the Worldwide Governance Indicator of country *j,* which reflects its economic and political stability.

##### Domestic economy index (*DE*)

Each step of the material’s value chain was based on the previous step. The upstream supply bottleneck directly affects or potentially threatens the entire value chain system of materials. In this case, this method considers the corresponding value of the material in each end-use to evaluate its importance to the national economy. The classification of end use refers to the China Statistical Yearbook (NBS. National Bureau of Statistics, 2019), but it is approximate as the accurate statistics are not available. More attention will be paid to the classification data to improve the reliability and accuracy of the evaluation results. In addition, the parameter indicating the characteristics of the material value endowed by the market is introduced. The market value of a certain material fluctuates with time, on which condition the average market value of each material in the past three months is selected as the calculation basis to define the importance of the material itself. The detailed calculation equation is as follows:

(Equation 5) $$DE_{M}=Q_{M}\frac{1}{GDP_{CN}}\sum_{1}^{i}V_{i}S_{i}$$

where *DE*_*M*_ is the domestic economy index of material *M*. *Q*_*M*_ is the indicator representing characteristics of material value. *Vi* is the value of the industry of domestic economy corresponding to end use i, which can be obtained from data published by the National Bureau of Statistics (NBS. National Bureau of Statistics, 2019).GDP_CN_ is the Gross Domestic Product of China.

##### Environmental risk index (*ER*)

The environmental impact of the material also directly affects the criticality. The environmental risk of a metal includes its toxicity grade, the proportion of waste discharge to the environment in the production process, and the impact of related environmental protection measures taken by various countries.

In the 12th Five Year Plan for the prevention and control of heavy metal pollution, mercury, hexavalent chromium, lead, cadmium, and arsenic were defined as comprising the first grade of heavy metals, and nickel, copper, zinc, silver, vanadium, manganese, cobalt, thallium and antimony were listed in the second grade of heavy metals (Ministry of Ecology and Environment, 2011). The potential toxicity of heavy metals is ranked according to their intrinsic chemical properties. Herein, the toxicity values of metals were qualitatively assigned as follows: the value of almost non-toxic metal was 0.1, the value of slightly toxic metal was 0.34, the value of toxic or very toxic metal was 0.67, and the value of extremely toxic or highly toxic metal was 1.

To evaluate the impact of waste discharged from the material processing stage on environmental risk, this method used the existing mainstream processing and smelting technology for the evaluation. The emission data were obtained from the manual of industrial pollution source emission coefficient (China, the State Council of the People's Republic of China, 2011) and the on-site investigation of influential enterprises in the domestic industry. However, because of the difficulty in obtaining the emission data, unavailable data were adopted and modified from the relevant research of Yale University (Graedel et al., 2015).

The corresponding environmental protection measures and policies issued by countries also directly affect the environmental risks of metal resources. The Environmental Performance Index (EPI, 2020) published by Yale University were used in this study. The indicator system established by the EPI focuses on the environmental sustainability and environmental performance of each country. The index ranks 163 countries based on 25 performance indicators, including the environment, public health, and ecosystem vitality (EPI, 2020).

The calculation method of environmental risk index of metals is as follows:

(Equation 6) $$ER_{M}=T_{M}（\frac{Q_{wg,M}}{Q_{wg}}+\frac{Q_{wl,M}}{Q_{wl}}+\frac{Q_{ws,M}}{Q_{ws}})EPI_{CN}$$

where *ER*_*M*_ refers to the environmental risk index of material *M*. *T*_*M*_ is the toxicity value of *M*. *EPI*_*CN*_ is the Environmental Performance Index for China. *Q*_*wg,M*_,*Q*_*wl,M*_, and *Q*_*ws,M*_ are the total amounts of waste gas, wastewater, and solid waste discharged in the production process per ton of product M, respectively. *Q*_*wg*_, *Q*_*wl*_, and *Q*_*ws*_ are the annual amounts of waste gas, wastewater, and waste solid emissions in China, respectively.

##### Criticality

Based on the above methodology, a large amount of data is collected and calculated to define the list of critical metals in China. In addition, expert consultation was used to assess accuracy. When both the supply safety index and domestic economy index of a certain material are very high, and the environmental risk index is located at a high degree, the material is defined as a critical metal. Critical metals are more difficult to obtain, and their sustainable supply will have a greater impact on China’s economy.

**Figure 3:**
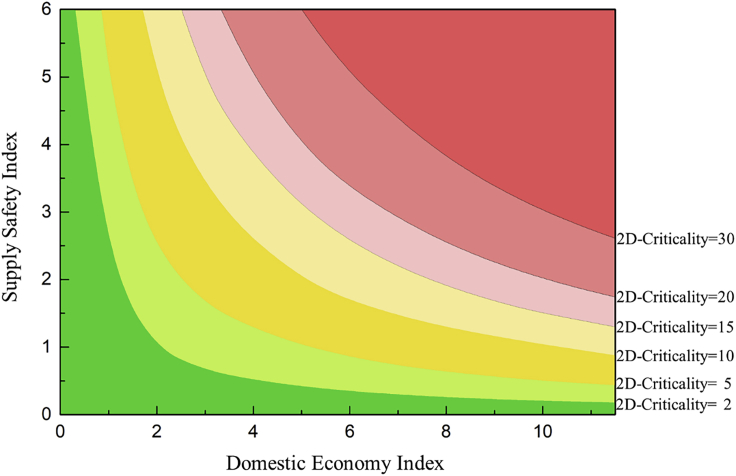
Two-dimensional matrix of 2D-Criticality metals

When the factors influencing supply safety and the domestic economy are considered, the criticality matrix of metals can be presented in the two-dimensional coordinate system with the domestic economy index as the x-axis and the supply safety index as the y-axis (Figure 3). In the two-dimensional matrix, the two-dimensional criticality (2D-Criticality) is defined as the product of the supply safety index and domestic economy index (Equation (7)), which is delimited by the contour lines marked in Figure 3. Metals located in the red and yellow areas (2D-Criticality ≥ 2), which represent a higher degree, require further evaluation. Materials with lower 2D-Criticality will be positioned in the green area (2D-Criticality < 2), implying a sustainable supply chain and less economic influence.

(Equation 7) $$2D-Criticality_{M}=SS_{M}*DE_{M}$$

**Figure 4:**
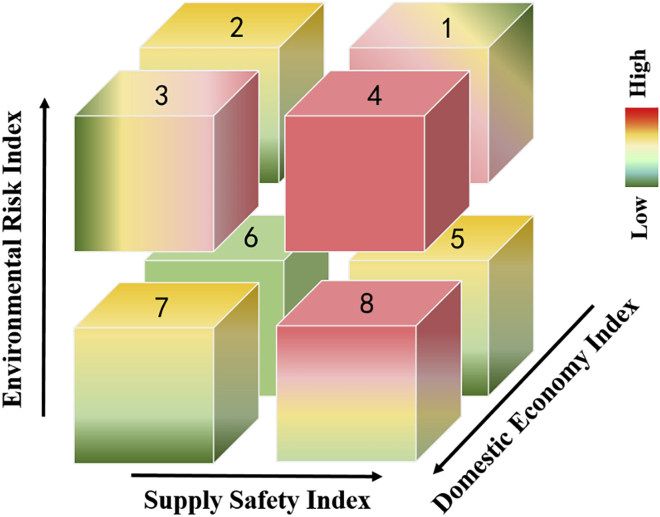
Three-dimensional representation of metal 3D-Criticality

When the comprehensive influences of the supply safety dimension, domestic economy dimension, and environmental risk dimension are considered, the material criticality (3D-Criticality) will be displayed in a three-dimensional space with the supply safety index as the x-axis, the domestic economy index as the y-axis, and the environmental risk index as the z-axis. As shown in Figure 4, quadrant 4 is the area with the highest 3D-Criticality, which is shown in red. Metals that fall in quadrant 4 are first-class critical metals. Quadrant 6 is the area where non-critical metals are located, with the values of the three dimensions for the evaluated metals being the lowest and marked in green. Regarding the other quadrants, the color gradually transits from red to yellow and then to green as the material 3D-Criticality changes from high to low.

### Data resources

The availability and quality of the adopted data should be guaranteed to ensure the accuracy and comparability of the results and maximize the quality of the outputs. In this study, a large number of data were required to complete the assessment. It is noteworthy that most data in the EC report (European Commission, 2017) (i.e., import/export amount, recycling information, consumption structure of resources, waste discharge) are not suitable for China. Therefore, the data are obtained through various data sources, and the priority order in this study is Chinese official data (e.g., National Bureau of Statistics and General Administration of Customs), industry reports issued by professional research institutions, data for China over global datasets (e.g., the United States Geological Survey and European Commission), authoritative journals, some data from non-official organizations or trade associations after checking reasonable, questionnaire survey directly from experts and relevant enterprises, best estimation, or expert judgment. This sequence also reflected the sensitivity coefficient. All data were normalized, and the index was dimensionless. The main data sources for each indicator in this study are listed in Table 1.

**Table 1:** Data sources used in this study

Index	Indicator	Data sources	In detail
Supply safety index	*SI*_*M*_ *SI*_*M,j*_	European Commission, 2014; European Commission, 2017	Table S1
	*Do* *Im* *Ex*	DOC, 2016; CBCIE, 2019; Chai et al, 2017; CHYXX, 2014; CHYXX, 2017a; CHYXX, 2017b; CHYXX, 2018a; CHYXX, 2018b; CHYXX, 2018c; CHYXX, 2019a; CHYXX, 2019b; CMRN, 2019; Futures, 2020; Lin, 2010; PISRI. Panzhihua Iron and Steel Research Institute, 2020; Liu et al, 2015; Long et al, 2016; Luo et al, 2017; Metalsinfo, 2019; Nengapp, 2019; Zou et al., 2017	Table S4
	*WGI*_*j*_	WGI, 2019	Table S5
	*HHI*_*WGI,M*_	USGS, 2020; WGI, 2019	Table S6
	*ρ*_*M*_	Graedel et al, 2011; USGS, 2020; UNEP, 2011 and information published by Chinese recycling companies	Table S4
Domestic economy index	*Q*_*M*_	SMM, 2020	Table S3
	*V*_*i*_	NBS. National Bureau of Statistics, 2019	Table S2
	*GDP*_*CN*_	NBS. National Bureau of Statistics, 2019	
Environmental risk index	*T*_*M*_	Qualitative assignment based on (Pradyot, 2002)	Table S7
	*Q*_*wg,M*_*; Q*_*wl,M*_*; Q*_*ws,M*_	China, the State Council of the People’s Republic of China, 2011	
	*Q*_*wg*_*; Q*_*wl*_*; Q*_*ws*_	Graedel et al., 2015; NBS. National Bureau of Statistics, 2019	Table S8
	EPI_CN_	EPI, 2020	

## Results and discussion

By making use of the methodology and detailed data described above, we evaluated the supply safety, domestic economy, and environmental risk dimensions for the 64 elements. The results are presented and discussed below.

### Supply safety index

According to (Equation 1), (Equation 2), (Equation 3), (Equation 4), the values of supply safety are calculated as shown in Table 2. The background color gradient (green to yellow to red) indicates changes in the supply safety (low to high). There are several metals with high scores in the supply safety dimension, indicating that they are more likely to face a high risk of supply shortage in the future. Boron, scandium, silicon alloy, vanadium, selenium, zirconium, and rare earth elements have high values due to low recovery rates. Lithium, chromium, platinum group metals, cobalt, niobium, germanium, nickel, and tantalum have high scores due to the heavy reliance on imports from foreign countries; molybdenum and rare earth elements have high substitutability indicators, that is to say, the characteristics of these metals are almost irreplaceable in their end uses, leading to their high SS; beryllium and tungsten are materials with high scores owing to the poor economic and political stability of the primary countries of production. It is worth noting that the SS for arsenic and mercury is not high, although the recycling rate is almost zero on the basis of the properties of toxicity and low market value. This is because the import reliance and substitutability indicators of arsenic and mercury are low. In addition, the HHI for gallium is very high, and the recovery rate is low, resulting in a tendency for high supply risk. However, a large proportion of gallium is produced for export in China, and its substitutability in end uses is high, so it is considered to have a medium supply security index. It is concluded that the supply safety dimensions of materials are determined by multiple factors rather than the performance of a single factor, which is the main cause of inaccuracies in current metal criticality evaluations in China.

**Table 2:** Evaluation results of indicators for resource supply safety indices

### Domestic economy index

**Figure 5:**
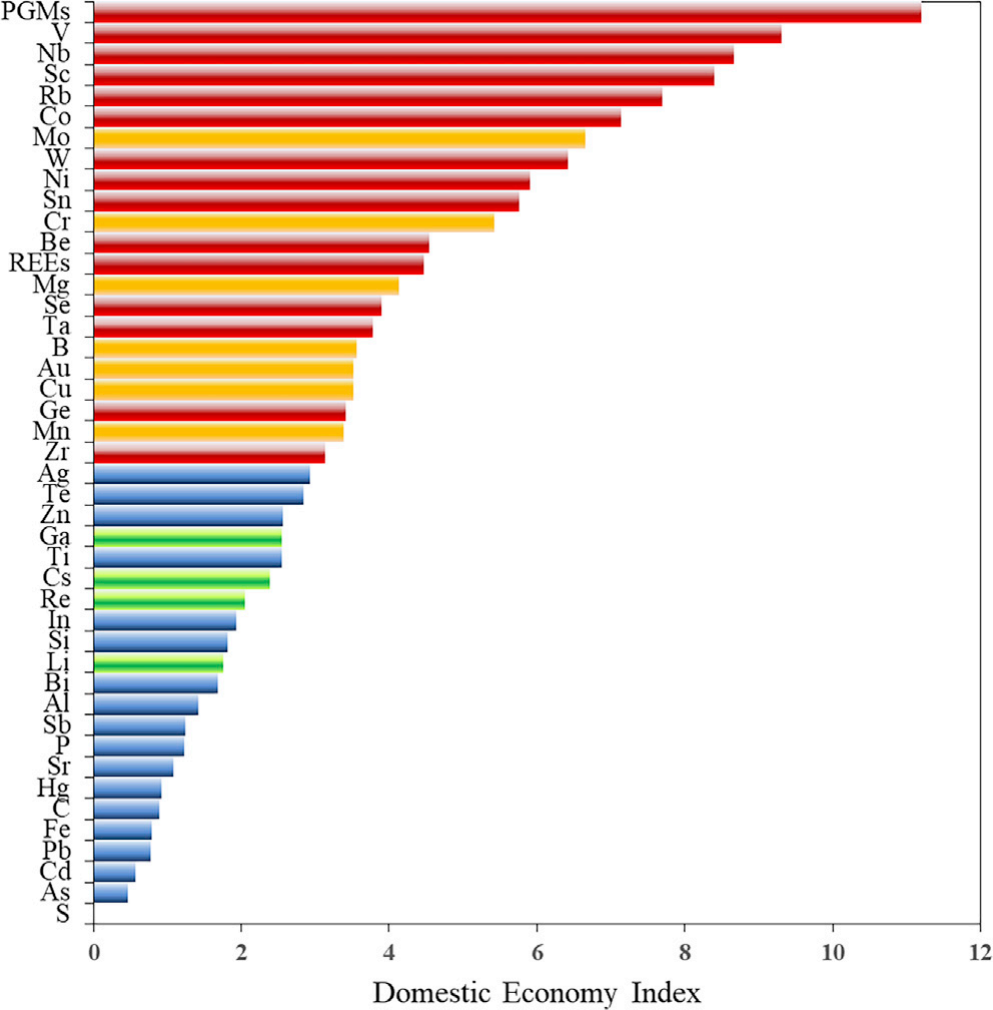
Evaluation results of domestic economy index for resources (Red bars refer to the metals with both high domestic economy index and high 3D-Criticality degree; metals with the yellow bar have high domestic economy index but low 3D-Criticality; metals with medium domestic economy index but identified as critical fall in the green area; metals in the blue area have both low domestic economy index and low criticality value).

According to the evaluation method of the domestic economy index listed in Section Approach, the related indicators of different metals were calculated, and the results are shown in Figure 5 and Table 3. The evaluation results represent the relative importance of metals to the Chinese national economy, ranging from high (e.g., platinum group metals, vanadium) to low (e.g., arsenic and sulfur). However, metals with a lower domestic economy index are not necessarily less important than those with higher index values. This parameter indicates that if the supply of the latter is restricted, it may have a greater potential impact on the economic value chain in China. Therefore, whenever a shortage of metals with a high domestic economy index occurs, it may hinder the strategic layout or development of high-tech technologies in China and other regions.

**Table 3:** Evaluation results of supply safety index, domestic economy index, and environmental risk index for resources

Figure 5 shows that the domestic economy results of several metals are inconsistent with the criticality degree due to the comprehensive influences of other dimensions. Although molybdenum and copper are of high economic importance, their potential supply risk in the future and the environmental impact are not significant; in this context, they are not identified as critical metals in three-dimensional assessment. Chromium, boron, magnesium, and manganese yielded high scores in both supply safety and domestic economy dimensions; however, with minor environmental impact, they were designated as non-critical metals. Heavy-polluted and high-value gold are not on the list of critical metals because their supply sustainability is expected to be stable in the future. Regarding gallium, cesium, rhenium, and lithium, the associated domestic economy indices are not high, and they are likely to face supply disruptions in the future, resulting in a high criticality degree.

### Environmental risk index

**Figure 6:**
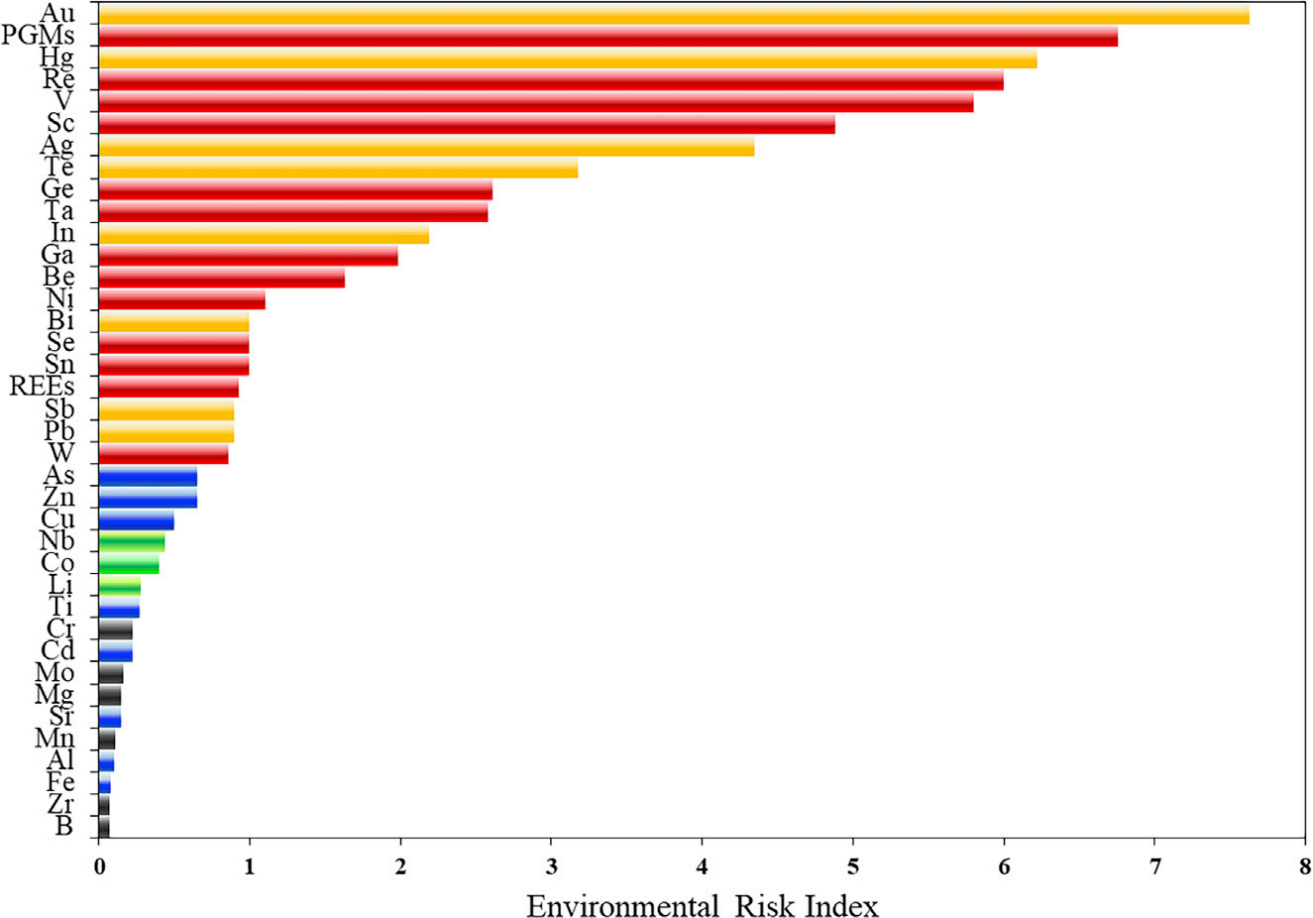
Evaluation results of environmental risk index for resources (Red bars refer to the metals with both high environmental risk and 3D-Criticality degree; yellow area represents metals with significant environmental problems but low 3D-Criticality value; metals with low environmental risk but high 3D-Criticality are marked in green bars; the blue bars represent metals that are neither critical nor noteworthy for environment; black bars are critical metals in two-dimensional but non-critical in three-dimensional assessment)

The toxicity grade of metals, the pollutants generated in the production process, and the environmental performance of each country are considered in the calculation of environmental risk to indicate the integrated impacts of metals on the environment. The results are shown in Figure 6 and Table 3. Rubidium and cesium and non-metallic resources of carbon, sulfur, phosphorus, and silicon were not included in the environmental risk assessment because of data source limitations. 3D-Criticality is a comprehensive reflection of the three evaluation dimensions. As shown in Figure 6, although some metals (e.g., gold, mercury, lead, etc.) have higher environmental risks, they are not evaluated as critical metals because of their stable supply or lower economic value. Although lithium and cobalt have relatively little impact on the environment, their potential supply risk is significant because of the rapid development of the new energy vehicle industry, which is also a critical metal for China’s industrial development. Owing to the high dependence of niobium on imports and its indispensable role in a number of end uses of tungsten, these metals are considered critical. In addition, chromium, molybdenum, manganese, magnesium, boron, and zirconium are critical metals in the two-dimensional evaluation, but they are non-critical metals in the three-dimensional assessment because of low scores in the environmental risk index.

### Criticality

#### Two-dimensional criticality assessment

**Figure 7:**
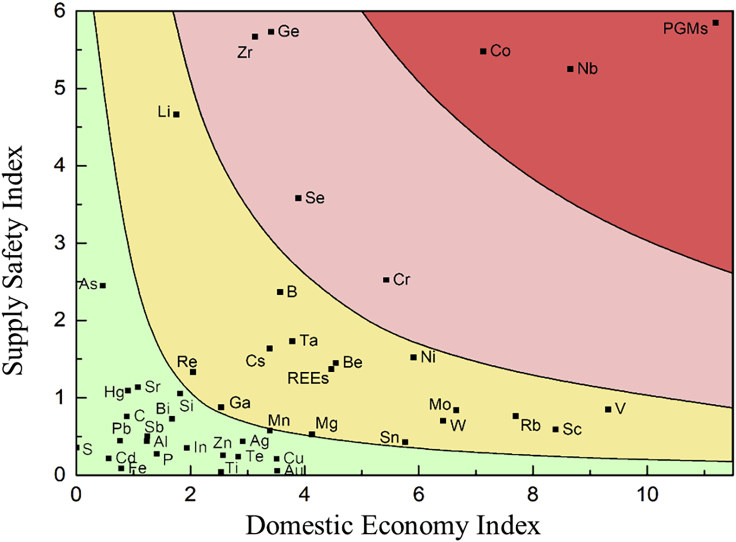
Criticality of different resources in the two-dimensional matrix

The results listed in Figure 7 and the detailed calculation data in the supplementary file A were derived from the above evaluation. The two-dimensional criticality (2D-Criticality) of metals is distributed in a two-dimensional coordinate system with the domestic economy dimension as the x-axis and the supply safety dimension as the y-axis. Three contour lines were identified in the matrix based on normalization and projection results. The farther the location is from the axis (in the upper right area of the graph), the higher the 2D-Criticality. The metals located in the dark red and light red areas of Figure 7 are designated as first-class critical materials, and materials with 2 <*SS*_*M*_*∗DE*_*M*_< 10 (deep yellow area of Figure 7) are determined to be second-class critical in the two-dimensional assessment. The materials positioned in the green area represent little effect and are identified as non-critical.

**Figure 8:**
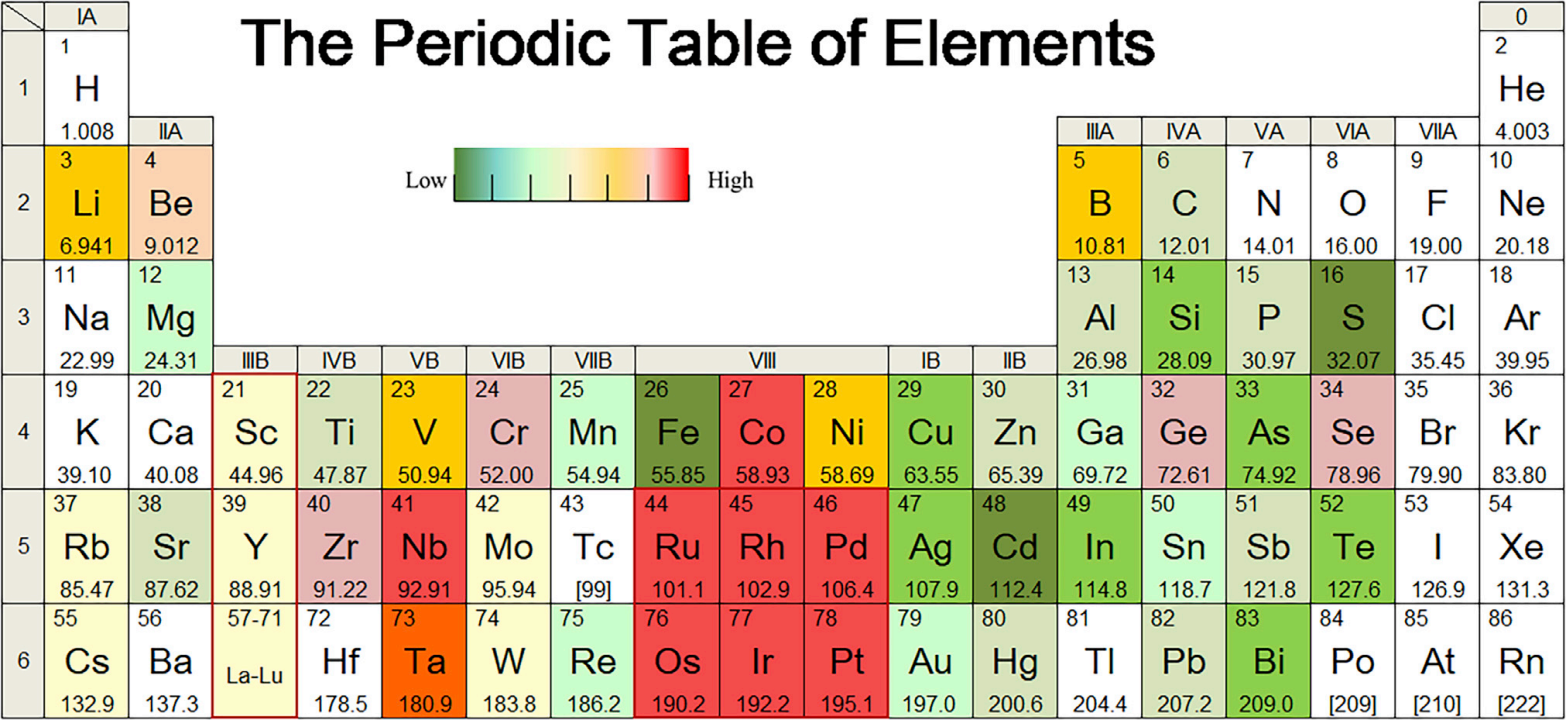
Distribution of 2D-Criticality for different resources in the periodic table of elements

As illustrated in Figure 7, the following critical metals were analyzed two-dimensionally: platinum group metals (PGMs), niobium, cobalt, germanium, selenium, chromium, zirconium, nickel, lithium, vanadium, molybdenum, boron, tantalum, cesium, rhenium, gallium, beryllium, manganese, magnesium, rare earth, tin, tungsten, rubidium, and scandium. PGMs comprise platinum, palladium, iridium, rhodium, ruthenium, and osmium. Rare earth elements include yttrium, scandium, and lanthanides (lanthanum, cerium, praseodymium, neodymium, promethium, samarium, europium, gadolinium, terbium, dysprosium, holmium, erbium, thulium, ytterbium, and lutetium). Based on the evaluation results of the 2D-Criticality, the color scales in the order of criticality are presented in the periodic table of elements, which can intuitively and clearly obtain the criticality distribution law of resources (Figure 8). The first-class critical metals located in the red area with two-dimensional assessment imply higher potential supply risk and greater economic influence, which require significant attention. The characteristics of these first-class critical metals are discussed in detail below.

PGMs: PGMs are regarded as expensive precious metals, with 90% being platinum and palladium (USGS, 2020). Platinum (40%) and palladium (80%) are consumed in the end use of catalysts (An and Ye, 2015). In addition to automotive catalysts, PGMs also play an important role in the proton exchange membranes of fuel cells (Reshetenko et al., 2016). The reserves of PGMs in China are quite poor, and production accounts for less than 1% of the world’s total production. PGMs can substitute for each other, but suitable substitutes for PGMs are unavailable (Richard, 2014). In this case, the design of new energy cells represented by fuel cells to reduce the application of PGMs, and the development of new alternative materials are effective measures for alleviating the supply crisis and reduce costs.

Niobium: Niobium is mainly used in steel production in the form of ferroniobium, and high temperature alloys made using some niobium compounds are used in the superconducting material industry (Hon et al., 2003), in which niobium is endowed with high industrial chain value. In addition, although China has the second largest niobium reserves in the world, its production only accounts for less than 1% of the production concentrated in Brazil and Canada (USGS, 2020), resulting in a large import reliance on niobium for China. The politics and economic stability of the main producing countries have a great influence on the supply safety of niobium owing to the high concentration of production. The main approach for alleviating the supply risk of niobium in China is to develop a new technology for processing niobium ore and improving its utilization rate.

Cobalt: Most cobalt use in China is in the preparation of battery materials as well as the synthesis of superalloys, cemented carbide, and magnetic materials (FREE, 2018), which are of great value to the emerging energy industry and manufacturing industry chain. The production of cobalt is concentrated in the Congo, Russia, and Australia, while reserves of cobalt in China are small (USGS, 2020). Therefore, the demand for cobalt is highly dependent on foreign countries. With the continuous development of the new energy industry, the supply shortage of cobalt will become increasingly prominent.

Germanium: The output of germanium in China ranks first globally, followed by Russia. The sum of the two countries accounts for 70% of the total amount worldwide (USGS, 2020). However, Chinese domestic production is far from meeting the demand for germanium. The production concentration of germanium is so high that the stability and policies in producing countries will significantly influence the global germanium market. Moreover, the end use of germanium-doped fiber has the advantages of large capacity, small light loss, low dispersion, long transmission distance, and lack of environmental interference (Hayashi et al., 1994). It is the only fiber that can be widely used on an engineering scale with no substitutes. Currently, the theoretical recovery rate of germanium is 30% (Graedel et al., 2011), which can be further improved by recycling technology advancement in the future.

Zirconium: The reserves and production of zirconium in China account for only 0.6% and 5% of the worldwide amount, respectively (USGS, 2020). Under these conditions, Chinese demand for zirconium is almost entirely imported from foreign countries. Conversely, the utilization ratio of secondary resources for zirconium is low, with a recycling rate of nearly zero. Therefore, the potential supply risk of zirconium in the future is likely to hinder the development of technologies related to its end uses.

Selenium: Selenium is often used to manufacture electronic devices (e.g., photocells, photosensitizers, and laser devices) owing to its photosensitivity and semiconductor properties (Takimiya and Otsubo, 2005), thus playing an indispensable role in the Chinese electronics industry. The addition of selenium to steel can significantly improve the mechanical and processing properties of steel (Coleman and Mccrosky, 2010). Selenium is usually associated with sulfide ores of copper in China, with its production accounting for 1/3 of the total global amount. Chinese consumption is far beyond its output. In the future, to address the gap between supply and demand for selenium in China, more effort should be dedicated to the technological development of selenium ore and secondary resources.

Chromium: 90% of chromium is used to prepare stainless steel, alloy steel, and non-ferrous alloys in the metallurgical industry (Mary and Lindsey, 1950), and the rest is consumed in preparing refractory and cast iron, which is of great significance to the Chinese iron and steel manufacturing industry. The production of chromium in China is negligible compared with the demand, with 95% of consumption driven by foreign countries. The recycling rate of chromium is less than 30%, which intensifies the contradiction between supply and demand. Effective measures to mitigate the chromium resource crisis include developing suitable substitutes for various industries as well as effective recycling technologies.

#### Three-dimensional criticality assessment

**Figure 9:**
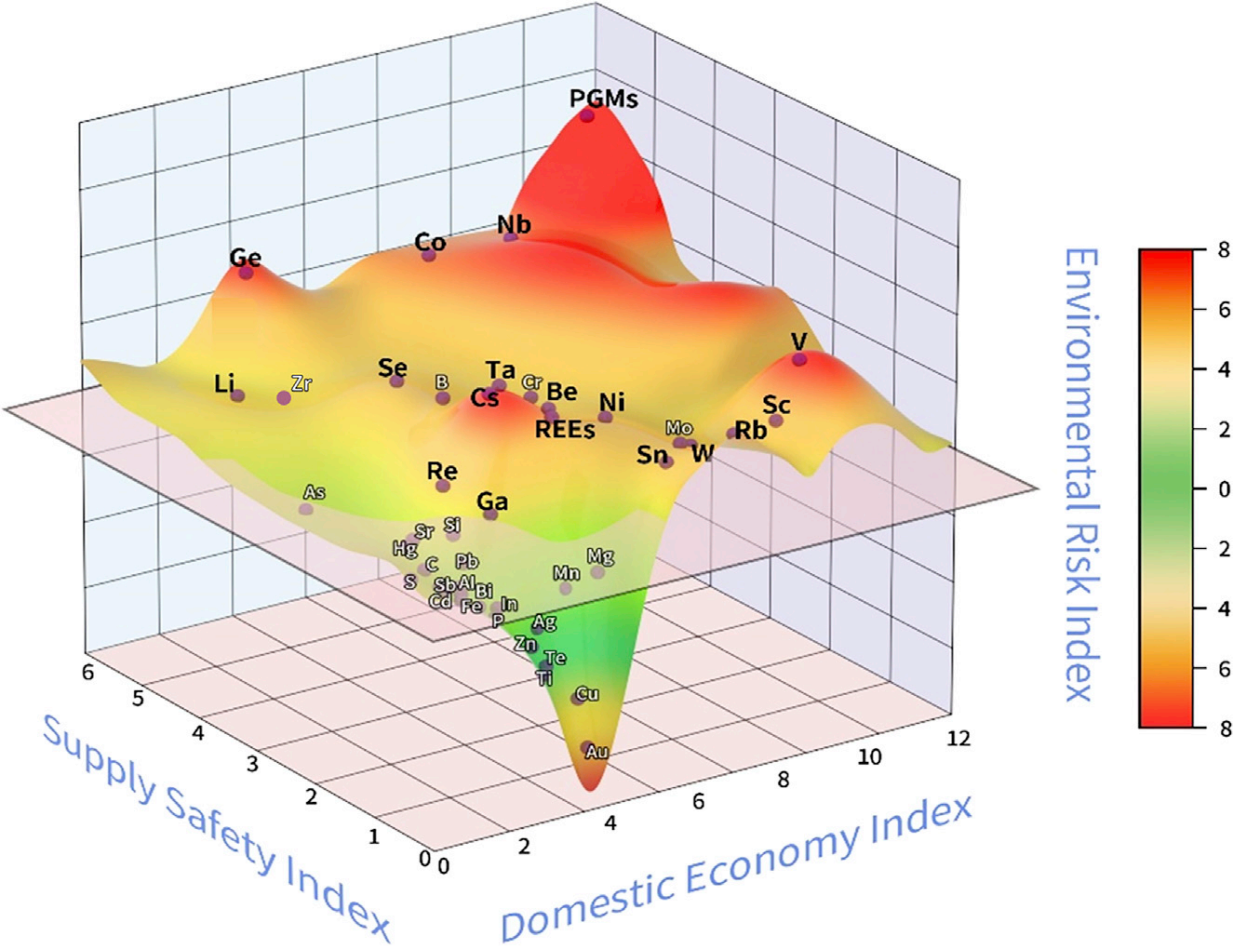
Criticality ofdifferent resources in the three-dimensional space (Critical metals are marked with black bold font; non-critical metals are marked with smaller white font)

Further information can be obtained when the influences of the supply safety dimension, domestic economy dimension, and environmental risk dimension are considered by plotting the results of three-dimensional assessment (3D-Criticality) in a three-dimensional criticality space (Figure 9). To show the results explicitly, metals with high 2D-Criticality (Figure 7) are concentrated in the rear area of Figure 9, where the environmental risk increases with the upward extension; metals with low 2D-Criticality fall in the front region, where the environmental risk increases with the downward extension. The color in Figure 9 (red to yellow to green) represents the environmental risk index (from large to small). In the upper back area of the graph, PGMs, niobium, and cobalt are the most critical metals with high supply risk and significant economic and environmental influences. The middle of the left rear area is favorable for suppliers, where the supply risk of metal products provided by suppliers in this region is high, which limits the number of competitors. Conversely, the middle of the right front area provides a region of opportunity for users, where the supply safety dimension of metals is not significant, indicating that the metal supply is available and sufficient in a period. Sellers would like to focus on the right rear middle area for the high possibility of making profit, where metals possess high economic value and low environmental risk. In addition, it is noteworthy that boron, manganese, molybdenum, magnesium, and chromium are non-critical metals in three-dimensional assessment with environmental risk scores below the threshold (environmental risk index = 0.25), although they were identified as critical in the two-dimensional criticality assessment.

### Discussion

**Figure 10:**
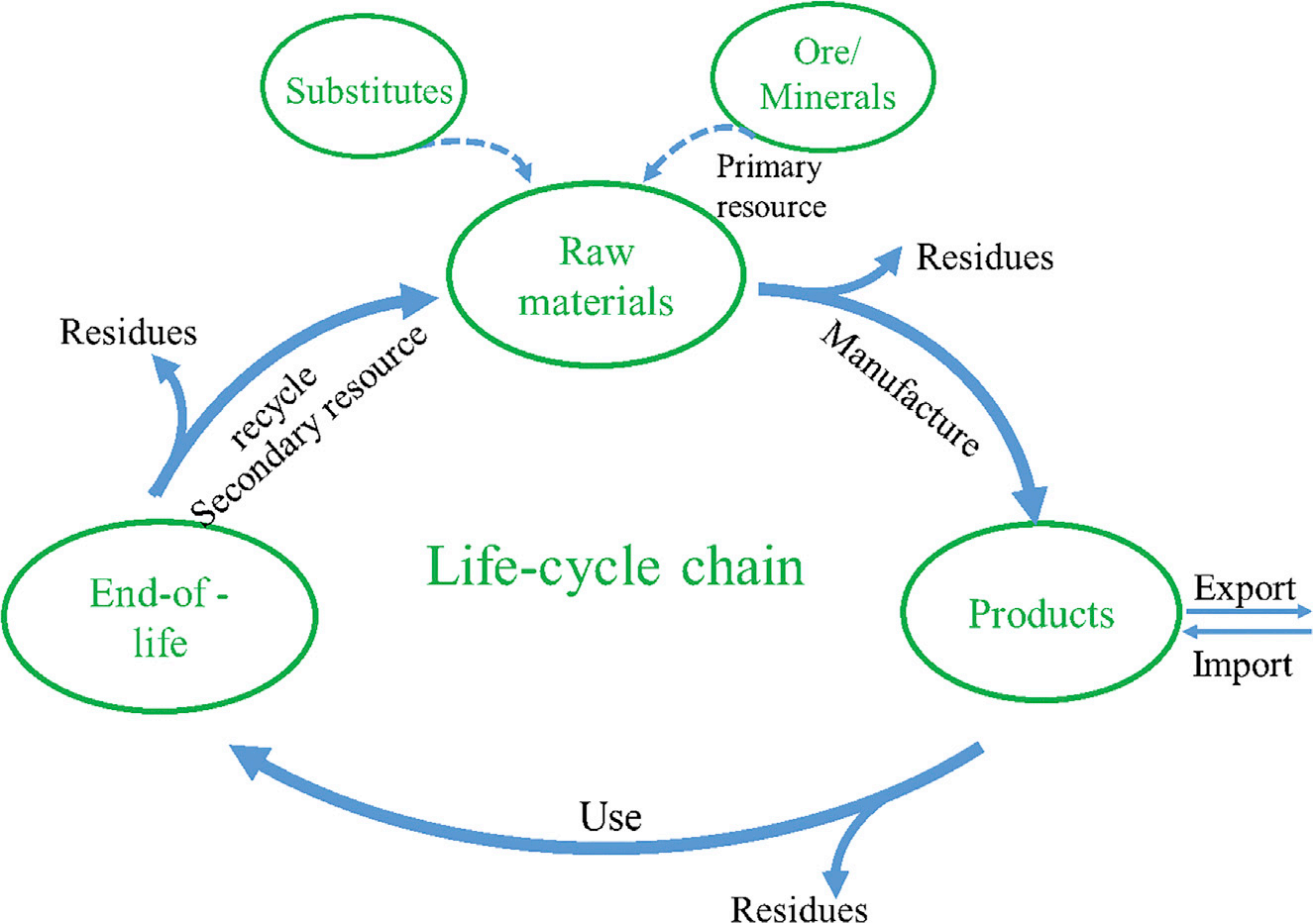
Conceptualization of the metal resources life cycle chain and circular economy

Identifying critical metals has attracted significant academic attention and provides important references for industrial markets and governmental decision makers. Generally, reducing the energy consumption of raw materials is emphasized in the design of industrial layouts (Gaustad et al., 2018). In the future, minimizing the criticality when choosing metals is of concern, especially for the long-term use of metals (Graedel and Nuss, 2014). The criticality assessment methodology established in this study integrates the design concept of the entire life cycle industrial chain and circular economy (Figure 10), in which the influence factors of each link in the entire process are fully examined when setting the evaluation indicators. The list of critical metals is the result of a comprehensive evaluation of supply safety and economic and environmental influences, while the results of every single dimension are also of great significance where certain bottlenecks with regard to each influence factor are clearly recognized. If the potential supply risk of metals is high due to the high dependence on imports, new technologies for primary resource utilization and recovery should be developed to improve the extraction rate to alleviate the risk, given that metal production distribution is highly concentrated. Paying close attention to the relevant technology progress and policies of the main supplier countries is key to addressing the potential supply constraints. Non-critical substitutes should be actively researched and developed when the substitutability of metals in the field is poor. Metals with high environmental risks cannot be ignored when formulating relevant policies and regulations by governments. The economic importance and environmental risk of metals should be simultaneously considered in adjusting and allocating the structure of industries or promulgating waste emission standards to promote enterprises to set up corresponding pollution disposal lines. In this context, the evaluation result of every indicator may provide a direct screen for designers and companies whose constant objective is to improve resource efficiency and availability and achieve beneficial outcomes in terms of both economic growth and ecological progress. For example, the main approaches are to reduce the use of critical metals by replacing them with non-critical substitutes, increasing utilization rates in manufacturing, and developing critical metal extraction and recycling technologies (Duclos et al., 2010). Enterprises or decision makers can adopt one or a combination of approaches to alleviate potential risks according to the assessment outcome.

### Limitations of study

In this study, a set of systematic and comprehensive criticality evaluation methodologies was established, but there are still some limitations. (1) The revolution and application of emerging technologies (*i.e.,* developing substitutes, advancement of primary resource extraction technology, optimization of secondary resource recovery technology) will significantly affect the supply and demand for metals, thus stimulating the rank of their criticality. In this case, the international situation of mineral resources and the balance between supply and demand of resources in China will be repositioned, which should be considered in future research. (2) In this methodology, the influences of national stability and relevant policy implementation on the criticality of materials are considered, whereas the corresponding parameters (i.e., HHI, WGI, and EPI) are updated once per year, which causes a reflection delay in which emergencies are unable to be identified in real time. For example, due to the global outbreak of COVID-19 at the beginning of 2020, countries have implemented various closed response measures, which necessarily limit international traffic and material flow. The supply security of metals has been significantly affected, and relevant data collection and evaluation are still in progress. (3) The calculation of the market value for metals in the domestic economy dimension adopted the classification of the national economy in the China Statistical Yearbook as an approximate estimate, and the classification corresponding to the end uses of metals was refined to optimize the results. The indicator representing the characteristics of material value has dynamic volatility and must be updated regularly with the market situation. (4) The assessments of supply safety and environmental risks were static. The future demand projections of materials and consumption life of mineral reserves can be supplemented in the parameter of supply sustainability risk to make the results more accurate and scientific. Although there are some limitations to this study due to the shortage of available data and complexity of evaluated factors, we believe that this methodology and the evaluation results are defendable, reliable, and significant. The criticality assessment and list of critical metals should be reexamined every 3-5 years with consistent data and compared with previous data.

### Conclusions

In this study, a systematic evaluation methodology for metal resource criticality was established. The influences of supply risk, economic fluctuation, and environmental risk are considered when setting parameters. In contrast to existing assessment methods, the sub-indicator setting of the supply safety dimension integrates the design concept of the entire life cycle industrial chain of raw materials. The dynamic value of metals evolving with the global market is elaborated in the domestic economy dimension, which establishes a close link between the static resource security situation and the ever-changing competitive market to help enterprises clarify trends of the metal market and formulate strategies to eliminate loss of interest. However, precisely determining metal criticality is highly complex. Although there are deficiencies in this method, we believe it to be informative and instructive.

Metal resource criticality was assessed using the established methodology in China for the first time, and a list of critical metals with great significance for China was obtained. When the influences of supply risk and economy fluctuation are considered, 24 metals are identified as critical in the two-dimensional coordinate system, whereas 18 metals have higher 3D-Criticality in the three-dimensional space with the environmental risk additionally considered. The values of platinum-group metals in the three dimensions are ranked in the first two places, indicating a higher supply shortage risk as well as a vital and fundamental role for the Chinese economy and facilities construction. The determination of critical metal resources provides a reference for China to alleviate the potential risk of resource supply, lays out emerging strategic technology industries, and actively responds to the international mining situation in the future.

We cannot assume that resources will always be available. The sustainability of resources in different fields is gradually becoming a more important topic. Only when the critical metals are taken seriously in the relevant industrial chain, economic market, and environmental management, can their sustainability be guaranteed. The methodology established in this work can be applied to the assessment of critical materials involved in specific areas such as lithium batteries, aircraft engines, and energy storage, which are the core and vital areas of worldwide attention and competition in the future. Moreover, the criticality of materials and technologies in various countries and regions can also be evaluated using this methodology when the relevant data are substituted and optimized.

## STAR★Methods

### Key resources table

**Table:** 

REAGENT or RESOURCE	SOURCE	IDENTIFIER
**Deposited data**		

Raw and analyzed data	List in Table 1	Presented in detail in the tables in supplymentary file

**Software and algorithms**		

Origin	N/A	Version 2019
Mathtype	N/A	Version 6.0
Matlab	MathWorks, Natick MA	Version 2018a

### Resource availability

#### Lead contact

Further information and requests for resources and reagents should be directed to and will be fulfilled by the lead contact, Zhi Sun (sunzhi@ipe.ac.cn).

#### Materials availability

This study did not generate new unique reagents.

#### Data and code availability

All data that supports the findings of this study are available from the corresponding author upon request and have been listed in detail in Supplementary File.

### Method details

#### Assessment scope

In this work, a comprehensive methodology for assessing criticality and applying the concept to raw materials selection. These assessments will help governments and enterprises take appropriate measures to mitigate supply restrictions, such as focusing more on critical metals in the economy and policy, actively promoting the research and development of extraction, recycling, and alternative materials of critical metals. This methodology can be applied to the assessment of critical materials involved in different areas and different regions. In this work, 64 elements in China were covered and criticality of raw materials for the next five years was evaluated and analyzed.

#### The basis and consideration of index selection

Based on the methodology of the European Commission, the parameter setting of the criticality assessment methodology established in this study comprehensively considers the multiple influences of many factors. The supply safety index integrates the design concept of the whole life cycle industrial chain and circular economy, in which the influence factors of each link in the whole process are fully examined when setting the evaluation parameters. The upstream supply of raw materials mainly comes from the production, import and export volume of China’s metal resources and the recyclable part from secondary resources. Besides, the emergence of substitutes will also affect the supply sustainability of critical metal resources. The global distribution of metal resources, the production concentration of suppliers, the economic and political stability of major suppliers and the promulgation of relevant policies will also have a great impact on resource supply. For example, since the 18th National Congress, China has attached great importance to the development of new energy vehicle industry. The “Energy-Saving and New Energy Vehicle Industry Development Plan (2012-2020)” issued by the State Council points out that by 2020, the cumulative production and sales of pure electric vehicles and plug-in hybrid electric vehicles will exceed 5 million. Subsequently, the new energy vehicle industry has entered the golden period of development, and the output and sales volume have increased rapidly. In 2016, the output was 519,000 units, and in 2018, the cumulative output was 1,222,000 units, with the assembly power battery being about 58.67 GWh. China has become a major producer and consumer of lithium-ion batteries in the world. With the rapid development of China’s new energy vehicle industry, the demand for nickel, cobalt, lithium, and other metal resources covered by power batteries has increased significantly, and the consumption structure of global related metal resources has changed accordingly. Great changes have taken place in the upstream industrial structure of lithium-ion battery related metal resources, which promotes the upgrading of resource extraction technology and accelerates the development and application progress of resource recovery technology. Meanwhile, the number and scale of resource recovery enterprises are rapidly expanded, and the exploration of alternative technology of raw materials is of concern. Therefore, this methodology sets up a supply safety index which comprehensively considers the influences of resources import reliance, secondary resource utilization, substitutability, distribution concentration, and stability of the supplier country to measure the supply risk of metal resources.

The criticality of resources is also closely related to market economic value. The real-time economic market is the feedback and wind vane of global metal resources and related industries. For example, after the introduction of relevant policies to promote the development of the global new energy vehicle industry, the prices of lithium and cobalt have increased significantly; it is stipulated in the Chinese new national standard that vanadium alloying elements must be added to rebar and the price of vanadium has risen by 100%, which has attracted great attention of the market. Hence, the parameter indicating characteristics of material value endowed by market is introduced. On the other hand, the supply bottleneck of upstream will directly affect or even threaten the whole value chain system of materials. In this case, this method takes into account the corresponding value of the material in each end-use to evaluate its importance to the national economy.

The future development layout of a country should take into account both economic construction and sustainable development of ecological environment. China’s economic industry is mainly heavy chemical industry with extensive development mode, which has caused a lot of waste of resources and serious environmental pollution. In order to promote the construction of an ecological civilization in China, it is necessary to clarify the impacts of toxicity of metal resources and pollutant emissions on the environment. In this context, the environmental risk index considering the toxicity of metal resources and the emission factors of three wastes in the production process is set to measure the environmental risks caused by the metal resource.

#### Dimensions of criticality assessment method

A three-dimensional system was designed, including the impacts of supply risk, economic fluctuation, and environmental risk.

#### Supply safety index

The supply safety index aims to characterize the supply risk of materials. It includes three aspects of risk: sustainability, reliance, and tolerance risk. The potential for one material to be replaced by another in its application field affects *SS*. The substitutability index is introduced in this method, which is the aggregate of the substitutability of materials in each application field. The degree of materials substitutability for each use is determined by experts in the industry (EU, 2013). If the substitutability value is 0, it means that it can be replaced at no cost; 0.3 indicates that it is feasible to be replaced at a lower cost; 0.7 means that it is feasible to be replaced with a high cost; and 1 indicates that it is irreplaceable. With the development of recycling technology, the supply of materials can come from not only primary resources but also secondary resources. If the recovery rate of a certain material is high, the contribution of secondary resources will be great, which can alleviate the supply risk in the future. The recovery rate of a certain material varies owing to the different waste products and technologies. Considering the reliability and unity of data sources, this study adopts the theoretical recovery rate of materials in published literature and reports (Graedel et al., 2011; USGS, 2020).

The dependence of material demand on foreign countries is also an important factor affecting *SS*. In this method, import reliance was introduced to indicate this effect, which is closely related to the production, consumption, import, and export of materials. The more we rely on imports, the greater the supply risk.

The Herfindahl-Hirschman index (HHI) is a comprehensive index to measure the degree of industrial concentration and has been widely used in anti-trust proceedings or assessments (European Commission, 2010). In this method, the HHI is used to evaluate the distribution concentration of metal resources. In the calculation of HHI, the economic and political stability of producing countries are also considered by involving the Worldwide Governance Indicator (WGI) published by the World Bank (WGI, 2019). If the value of HHI is high, the stability of the producing country will have a significant impact on the international market of the material. Emergencies in these countries will bring about supply risks.

The calculation formulas of supply safety index are described below:

(Equation 1) $$SS_{M}=SI_{M}TR_{M}\left(1-\rho_{M}\right)HHI_{WGI,M}$$

where *SS*_*M*_ represents the supply safety index of material M. *ρ*_*M*_ is the recycling rate of *M*. *SI*_*M*_ is the aggregate substitutability of *M*, and its calculation method is shown in Equation (2).

(Equation 2) $$SI_{M}=\sum_{1}^{\text{i}}SI_{M,\text{i}}S_{i}$$

where i represents the end-use of material *M. S*_*i*_ refers to the proportion of *M* in end-use I, and $\sum S_{i}=1$. *SI*_*M,i*_ *is the* substitutability of *M* in end-use i.

(Equation 3) $$TR_{M}=\frac{Do+Im-Ex}{Do}$$

*TR*_*M*_ refers to the import reliance of material *M* and can be calculated using Equation (3). *Do* is the annual domestic production of material *M* in China. *Im* and *Ex* are the annual import and export volumes of material *M* in China, respectively.

(Equation 4) $$HHI_{WGI,M}=\sum {S_{M,j}}^{2}*WGI_{j}$$

The calculation formula for HHI is shown in Equation (4), where *S*_*M,j*_ is the production percentage of material *M* produced by country *j* in the total global production of *M*. *WGI*_*j*_ is the Worldwide Governance Indicator of country *j,* which reflects its economic and political stability.

### Domestic economy index

Each step of the materials value chain was based on the previous step. The upstream supply bottleneck directly affects or potentially threatens the entire value chain system of materials. In this case, this method considers the corresponding value of the material in each end-use to evaluate its importance to the national economy. The classification of end-use refers to the China Statistical Yearbook (NBS. National Bureau of Statistics, 2019), but it is approximate as the accurate statistics are not available. More attention will be paid to the classification data to improve the reliability and accuracy of the evaluation results. In addition, the parameter indicating the characteristics of the material value endowed by the market is introduced. The market value of a certain material fluctuates with time, on which condition the average market value of each material in the past three months is selected as the calculation basis to define the importance of the material itself. The detailed calculation equation is as follows:

(Equation 5) $$DE_{M}=Q_{M}\frac{1}{GDP_{CN}}\sum_{1}^{i}V_{i}S_{i}$$

where *DE*_*M*_ is the domestic economy index of material *M*. *Q*_*M*_ is the indicator representing characteristics of material value. *Vi* is the value of the industry of domestic economy corresponding to end-use i, which can be obtained from data published by the National Bureau of Statistics (NBS. National Bureau of Statistics, 2019).GDP_CN_ is the Gross Domestic Product of China.

The environmental impact of the material also directly affects the criticality. The environmental risk of a metal includes its toxicity grade, the proportion of waste discharge to the environment in the production process, and the impact of related environmental protection measures taken by various countries.

In the 12th Five Year Plan for the prevention and control of heavy metal pollution, mercury, hexavalent chromium, lead, cadmium, and arsenic were defined as comprising the first grade of heavy metals, and nickel, copper, zinc, silver, vanadium, manganese, cobalt, thallium and antimony were listed in the second grade of heavy metals (Ministry of Ecology and Environment, 2011). The potential toxicity of heavy metals is ranked according to their intrinsic chemical properties. Herein, the toxicity values of metals were qualitatively assigned as follows: the value of almost non-toxic metal was 0.1, the value of slightly toxic metal was 0.34, the value of toxic or very toxic metal was 0.67, and the value of extremely toxic or highly toxic metal was 1.

### Environmental risk index

To evaluate the impact of waste discharged from the material processing stage on environmental risk, this method used the existing mainstream processing and smelting technology for the evaluation. The emission data were obtained from the manual of industrial pollution source emission coefficient (China, the State Council of the People’s Republic of China, 2011) and the on-site investigation of influential enterprises in the domestic industry. However, because of the difficulty in obtaining the emission data, unavailable data were adopted and modified from the relevant research of Yale University (Graedel et al., 2015).

The corresponding environmental protection measures and policies issued by countries also directly affect the environmental risks of metal resources. The Environmental Performance Indexes (EPI, 2020) published by Yale University were used in this study. The indicator system established by the EPI focuses on the environmental sustainability and environmental performance of each country. The index ranks 163 countries based on 25 performance indicators, including the environment, public health, and ecosystem vitality (EPI, 2020).

The calculation method of environmental risk index of metals is as follows:

(Equation 6) $$ER_{M}=T_{M}（\frac{Q_{wg,M}}{Q_{wg}}+\frac{Q_{wl,M}}{Q_{wl}}+\frac{Q_{ws,M}}{Q_{ws}})EPI_{CN}$$

where *ER*_*M*_ refers to the environmental risk index of material *M*. *T*_*M*_ is the toxicity value of *M*. *EPI*_*CN*_ is the environmental performance index for China. *Q*_*wg,M,*_*Q*_*wl,M*,_ and *Q*_*ws,M*_ are the total amount of waste gas, wastewater, and solid waste discharged in the production process per ton of product M, respectively. *Q*_*wg*_, *Q*_*wl*_, and *Q*_*ws*_ are the annual amounts of waste gas, wastewater, and waste solid emissions in China, respectively.

When the factors influencing supply safety and the domestic economy are considered, the criticality matrix of metals can be presented in the two-dimensional coordinate system with the domestic economy index as the x-axis and the supply safety index as the y-axis. In the two-dimensional matrix, the two-dimensional criticality (2D-Criticality) is defined as the product of the supply safety index and domestic economy index (Equation 7). Metals located in the red and yellow areas (2D-Criticality ≥ 2), which represents a higher degree, require further evaluation. Materials with lower 2D-Criticality will position in the green area (2D-Criticality < 2), implying a sustainable supply chain and less economic influence.

(Equation 7) $$2D-Criticality_{M}=SS_{M}*DE_{M}$$

When the comprehensive influences of the supply safety dimension, domestic economy dimension, and environmental risk dimension are considered, the material criticality (3D-Criticality) will be displayed in a three-dimensional space with the supply safety index as the x-axis, the domestic economy index as the y-axis, and the environmental risk index as the z-axis. The color gradually transits from red to yellow and then to green as the material 3D-Criticality changes from high to low.

## References

[bib1] An F., Ye X.H. (2015). Research on recycling platinum group metals from waste metal-based automobile catalyst. Inorg. Chem. Industry.

[bib2] Bach V., Finogenova N., Berger M., Winter L., Finkbeiner M. (2017). Enhancing the assessment of critical resource use at the country level with the scarce method – case study of Germany. Resour. Policy.

[bib3] Blengini G., Blagoeva D., Dewulf J., Torres D.M.C., Nita V., Vidal L., Latunussa C., Kayam Y., Talens P.,L., Baranzelli C. (2017). Assessment of the methodology for establishing the EU list of critical raw materials : annexes. https://publications.jrc.ec.europa.eu/repository/handle/JRC107008.

[bib4] Bram B., Henrike S. (2011). Critical thinking about critical minerals - assessing risks related to resource security. http://www.bgr.bund.de/DE/Themen/Min_rohstoffe/Downloads/Polinares_Critical_Thinking.pdf?__blob=publicationFile&v=3.

[bib5] CBCIE (2019). World and national refined tin consumption in 2019. http://www.cbcie.com/data/819107.html.

[bib6] Chai L., Bao Q.Z., Zhou Y.H., Li X. (2017). Resources and supply＆demand situation of precious metal in northeastern asia area. Conservation Utilization Mineral.Resour..

[bib7] China, Ministry of Land and Resources of the People’s Republic of China (2016). China’s list of strategic mineral list. http://www.gov.cn/xinwen/2016-11/30/content_5140509.htm.

[bib8] China, Ministry of Natural Resources of the People's Republic of China (2020). China mineral resources 2020. http://www.mnr.gov.cn/sj/sjfw/kc_19263/zgkczybg/202010/t20201022_2572964.html.

[bib9] China, the State Council of the People’s Republic of China (2011). Manual of Production and Discharge Coefficients of Industrial Pollution Sources.

[bib10] China, the State Council of the People’s Republic of China (2016). 13th Five-year plan for ecological and environmental protection. http://www.gov.cn/zhengce/content/2016-12/05/content_5143290.htm.

[bib11] China, the State Council of the People’s Republic of China (2016). Chinese national plan for mineral resource (2016-2020). http://www.mnr.gov.cn/gk/ghjh/201811/t20181101_2324927.html.

[bib12] CHYXX (2014). Analysis of market demand and competition of selenium industry. https://www.chyxx.com/industry/201404/235675.html.

[bib13] CHYXX (2017). Analysis of China's graphite output, consumption and import and export. http://www.chyxx.com/industry/201707/544446.html.

[bib14] CHYXX (2017). Analysis on the consumption and development trend of China's aluminum industry. http://www.chyxx.com/industry/201704/515454.html.

[bib15] CHYXX (2018). Analysis of sulfur production, import and export and price trend in China. http://www.chyxx.com/industry/201806/648536.html.

[bib16] CHYXX (2018). Analysis of the output and productivity of nickel ore in China. http://www.chyxx.com/industry/201806/653844.html.

[bib17] CHYXX (2018). China's cadmium production and consumption trend in 2017. http://www.chyxx.com/industry/201809/674908.html.

[bib18] CHYXX (2019). Analysis of global and Chinese tungsten production and consumption in 2018-2019. http://www.chyxx.com/industry/201909/788599.html.

[bib19] CHYXX (2019). Capacity distribution and market demand analysis of global and Chinese molybdenum industry in 2019. https://www.chyxx.com/industry/202001/827090.html.

[bib20] Claudiu C.P., Alain M., Patricia A.D., Darina B., Evangelos T. (2016). Substitution of Critical Raw Materials in Low-Carbon Technologies: Lighting, Wind Turbines and Electric Vehicles. https://publications.jrc.ec.europa.eu/repository/bitstream/JRC103284/crm%20substitution_online%20report.pdf.

[bib21] CMRN (2019). High purity arsenic market analysis report in 2019. http://www.cmrn.com.cn/show_7/35026.html.

[bib22] Coleman W.C., Mccrosky C.R. (2010). Determination of selenium in steel. J. Polym. ence.

[bib23] DOC (2016). The situation of 16 strategic mineral resources in the world. http://www.360doc.Com/Content/16/0730/08/7522678_579482240.Shtml.

[bib24] DOE.U.S. Department of Energy (2010). Critical materials strategy. https://www.energy.gov/eere/amo/2010-critical-materials-strategy.

[bib25] DOE.U.S. Department of Energy (2011). Critical materials strategy. https://www.energy.gov/node/349057.

[bib26] DOE.U.S. Department of Energy (2019). Sustainability report and implementation plan. https://www.energy.gov/sites/prod/files/2019/11/f68/DOE%202019%20Sustainability%20Report%20and%20Implementation%20Plan.pdf.

[bib27] Duclos S.J., Otto J.P., Konitzer D.G. (2010). Design in an era of constrained resources. Mech. Eng..

[bib28] European Commission (2010). Critical raw materials for the EU - report of the Ad-hoc Working Group on defining critical raw materials. https://www.mmta.co.uk/wp-content/uploads/2017/02/EU-Raw-Materials-Initiative-full-paper-Jun-10.pdf.

[bib29] European Commission (2014). Report on critical raw materials for the EU, Report of the Ad hoc Working Group on defining critical raw materials. http://ec.europa.eu/DocsRoom/documents/10010/attachments/1/translations/en/renditions/pdf.

[bib30] European Commission (2017). Study on the review of the list of critical raw materials. review of the list of critical raw materials - criticality assessments. https://publications.europa.eu/en/publication-detail/-/publication/08fdab5f-9766-11e7-b92d-01aa75ed71a1/language-en.

[bib31] European Commission (2020). Critical raw materials resilience: charting a path towards greater security and sustainability. https://eur-lex.europa.eu/legal-content/EN/TXT/?uri=CELEX:52020DC0474#footnote8.

[bib32] Eggert, Roderick G. (2011). Minerals go critical. Nat. Chem..

[bib33] Eheliyagoda D., Zeng X.L., Li J.H. (2020). A method to assess national metal criticality: the environment as a foremost measurement. Humanit.Social Sci. Commun..

[bib34] EPI (2020). Environmental performance index. https://epi.yale.edu/epi-results/2020/component/epi.

[bib37] Fortier S.M., Nassar N.T., Lederer G.W., Brainard J., Gambogi J., McCullough E.A. (2018). Federal register: draft list of critical minerals. https://pubs.er.usgs.gov/publication/ofr20181021.

[bib38] FREE (2018). Report on consumption frame and demand increase of Chinese cobalt in 2018. http://free.chinabaogao.com/yejin/201804/04163302142018.html.

[bib39] Fu X.K., Polli A., Olivetti1 E. (2018). High-resolution insight into materials criticality: quantifying risk for by-product metals from primary production. J. Ind. Ecol..

[bib40] Futures (2020). Analysis of copper reserves, production, consumption and consumption structure in China. http://futures.eastmoney.com/a/202001111354186014.html.

[bib41] Gardner L., Colwill J. (2018). A framework and decision support tool for improving value chain resilience to critical materials in manufacturing. Prod. Manuf.Res..

[bib42] Gaustad G., Krystofik M., Bustamante M., Badami K. (2018). Circular economy strategies for mitigating critical material supply issues. Resour. Conserv.Recycl..

[bib43] Graedel T.E., Allwood J., Birat J., Matthias B., Christian H., Barbara K.R., Scott F.S., Guido S. (2011). What do we know about metal recycling rates?. J. Ind. Ecol..

[bib44] Graedel T.E., Rachel B., Chelsea C., Thomas C., Joanne C., Lee C., Elizabeth F., Claire H., Christine J., Nedal T.N. (2012). Methodology of metal criticality determination. Environ. Sci. Technol..

[bib45] Graedel T.E., Harper E.M., Nassar N.T., Nuss P., Reck B.K. (2015). Criticality of metals and metalloids. Proc. Natl. Acad. Sci. U S A.

[bib46] Graedel T.E., Nuss P. (2014). Employing considerations of criticality in product design. JOM.

[bib48] Hatayama H., Tahara K. (2015). Criticality assessment of metals for Japan's resource strategy. Mater. Trans..

[bib49] Hayashi Y., Okuda Y., Mitera H., Kato K. (1994). Formation of drawing- or radiation-induced defects in germanium-doped silica core optical fiber. Jpn. J. Appl. Phys..

[bib50] Hayes S.M., McCullough E.A. (2018). Critical minerals: a review of elemental trends in comprehensive criticality studies. Resour. Policy.

[bib51] Helbig C., Schrijvers D., Hool A. (2021). Selecting and prioritizing material resources by criticality assessments. One Earth.

[bib52] Hon Y.H., Wang J.Y., Pan Y.N. (2003). Composition/phase structure and properties of titanium-niobium alloys. Mater.Trans..

[bib54] Ioannidou D., Heeren N., Sonnemann G., Habert G. (2019). The future in and of criticality assessments. J. Ind. Ecol..

[bib56] Ju J.H., Wang Q., Chen J.B. (2019). Study on the high quality development of China mining industry in the new era. China Mining Mag..

[bib58] Lin H.C. (2010). Status and development prospects of domestic metal scandium material. China Nonferrous Metall..

[bib59] Liu X., Chen Q.S., Zhang Y.F., Gao T.M. (2015). Chinese chromium demand forecasting and resource supply security. Resour. Sci..

[bib60] Long T., Chen Q.S., Yu W.J., Yu Q., Zhang Y.S. (2016). The analysis and suggestions of the bismuth's supply and demand in China. China Mining Mag..

[bib61] Luo Y.J., Wang X.L., Liu Q.Y., Chen Q.S., Zhou Y.J. (2017). The future demand of antimony in China. China Mining Mag..

[bib63] Marscheider-Weidemann F., Langkau S., Hummen T., Erdmann L., Tercero E., Angerer G., Marwede M., Benecke S. (2016). Raw Materials for Future Technologies (In German). http://refhub.elsevier.com/S0959-6526(20)30766-6/sref34.

[bib64] Mary B., Lindsey W.M. (1950). Chromium Stainless Steels.

[bib66] Metalsinfo (2019). Chinese Zinc Consumption Will Peak in the Middle of the Next Decade. https://www.metalsinfo.com/news/display_pid_9-cid_16-news_id_215597.html.

[bib65] Ministry of Ecology and Environment (2011). The 12th Five Year Plan for the Prevention and Control of Heavy Metal Pollution. http://www.gov.cn/xinwen/2016-11/30/content_5140517.htm.

[bib67] NBS.National Bureau of Statistics (2019). China Statistical Yearbook.

[bib68] Nengapp (2019). Prospect of indium resource demand in China. https://www.nengapp.com/news/detail/2433248.

[bib69] PISRI. Panzhihua Iron and Steel Research Institute (2020). Recent technological development and price trend of vanadium products. VanadiumTitanium News.

[bib70] Pradyot P. (2002). Handbook of Inorganic Chemicals.

[bib71] Rabe W., Kostka G., Smith Stegen K. (2017). China's supply of critical raw materials: risks for Europe's solar and wind industries?. Energy Policy.

[bib72] Reshetenko T., Serov A., Artyushkova K., Matanovic I., Stariha S., Atanassov P. (2016). Tolerance of non-platinum group metals cathodes proton exchange membrane fuel cells to air contaminants. J. Power Sources.

[bib73] Richard J.G. (2014). Critical metals handbook. Econ. Geol..

[bib74] Schrijvers D., Hool A., Blengini G.A., Chen W.Q., Wger P.A. (2020). A review of methods and data to determine raw material criticality. Resour. Conserv.Recycl..

[bib75] SETIS. Strategic Energy Technologies Information System, European Commission (2015). Securing Europe’s Critical Raw Material Supply Chain: The Role of Recycling. https://setis.ec.europa.eu/publications/setis-magazine/materials-energy/setis-feature-article-securing-europe%E2%80%99s-critical-raw.

[bib76] SMM (2020). Price of metal resources. https://www.smm.cn/.

[bib77] Song J.L., Yan W.Y., Cao H.B., Song Q.B., Ding H., Lv Z., Zhang Y., Sun Z. (2019). Material flow analysis on critical raw materials of lithium-ion batteries in China. J. Clean. Prod..

[bib78] Takimiya K., Otsubo T. (2005). Selenium-containing π-conjugated compounds for electronic molecular materials. Cheminform.

[bib53] US Department of the Interior (2018). Federal Register: Draft List of Critical Minerals. http://refhub.elsevier.com/S0921-3449(19)30523-3/sbref0540.

[bib79] USGS (2020). Mineral commodity Summaries. https://www.usgs.gov/centers/nmic/mineral-commodity-summaries.

[bib80] Wang D.F., Wang W.J., Chen W.Q. (2019). Supply security of strategic metal ores in China. Resources Industries.

[bib81] WGI (2019). Worldwide governance indicators. http://data.worldbank.org/datacatalog/worldwide-governance-indicators.

[bib82] Yan W.Y., Cao H.B., Zhang Y., Ning P.G., Song Q.B., Yang J.X., Sun Z. (2020). Rethinking Chinese supply resilience of critical metals in lithium-ion batteries. J. Clean. Prod..

[bib83] Zou Y., Shi D.F., Zeng L.Y. (2017). China's mercury resource development and management under the new situation. China Mining Mag..

[bib85] https://www.resourcepanel.org/reports/recycling-rates-metals.

[bib86] 2014. http://ec. europa.eu/DocsRoom/documents/10010/attachments/1/translations/en/ renditions/pdf.

