# Supplementary material for: Criticality assessment of metal resources in China
Source: iScience. 2021 May 8;24(6):102524. doi: 10.1016/j.isci.2021.102524 (PMC8188489; doi:10.1016/j.isci.2021.102524)
Supplement: Document S1. Tables S1–S8 [file mmc1.pdf]

iScience, Volume *24*

Supplemental information

Criticality assessment

# of metal resources in China

Wenyi Yan, Zhaolong Wang, Hongbin Cao, Yi Zhang, and Zhi Sun

Supplementary Data:

Table S1 related to Table 1. Consumption structure, end-use value and substitution of resources in China

Table S2 related to Table 1. Main industries of national economy and total economic value

Table S3 related to Table 1. Results of domestic economy index and characteristics value parameter of resources

Table S4 related to Table 1. The recycling rate and import reliance of resources

Table S5 related to Table 1. World government index for countries

Table S6 related to Table 1. Chinese production share and Herfindahl-Hirschman index of resources

Table S7 related to Table 1. Toxicity degree value of metals

Table S8 related to Table 1. Annual amount of waste gas, waste water and waste solid emission in China and Environmental Performance Index for China

Detailed data for the supply safety index, domestic economy index and environmental index. Data sources are in Table 1 in the manuscript.

Table S1. Consumption structure, end-use value and substitution of resources in China

**Resource**	**Application**	**Share (S_i)**	**Value (S_i*V_i)**	**Value added**	Substitutability ($SI_{M,i}$)	Substitutability **($SI_{M,j*}$ S_i)**	SI_M
**Alumium（Al）**	Transport	22%	8177.97	83381.45	0.7	0.154	0.56
	Building	34%	18806.69		0.5	0.17	
	Packaging	11%	26455.59		0.5	0.055	
	Consumer goods	8%	6212.66		0.5	0.04	
	Machinery manufacturing	8%	19240.43		0.7	0.056	
	Electronics & communication	17%	4488.10		0.5	0.085	
**Chromium（Cr）**	Metallurgy (stainless steel, alloy steel, alloy)	90%	216454.86	232363.04	1	0.9	1
	Refractory and cast iron	5%	12025.27		1	0.05	
	Chemicals	5%	3882.91		1	0.05	
**Cobalt**	Batteries	77%	185189.16	217702.32	0.8	0.616	0.777

**（Co）**	Hard Materials - Carbides, Diamond Tooling & Other Alloys	8%	19240.43		0.7	0.056	
	Magnets	4%	9620.22		0.7	0.028	
	Superalloy	4%	2212.55		0.7	0.028	
	Catalysts	3%	792.02		0.7	0.021	
	Others	4%	647.94		0.7	0.028	
**Copper（Cu）**	Building	2%	1106.28	149513.76	0.5	0.01	0.548
	Electrical infrastructure and equipment	53%	127467.86		0.5	0.265	
	Transport	9%	3345.53		0.5	0.045	
	Air conditioning, refrigerator	12%	9318.98		0.5	0.06	
	Electronics & ICT	6%	1584.04		0.7	0.042	
	Others	18%	6691.07		0.7	0.126	
**Gallium**	Optoelectronic devices	41%	10824.25	64629.66	0.7	0.287	0.632

**（Ga）**	Integrated circuits	25%	6600.15		0.7	0.175	
	Alloy, medicine, others	17%	40885.92		0.5	0.085	
	Solar	17%	6319.34		0.5	0.085	
**Indium （In)**	Flat panel displays	70%	18480.42	51481.05	0.7	0.49	0.688
	Compound semiconductors & LEDs	12%	3168.07		0.7	0.084	
	Solders, alloys	12%	28860.65		0.7	0.084	
	Others	6%	971.91		0.5	0.03	
**Iron （Fe）**	Steel: Construction	44%	24338.07	79130.22	1	0.44	0.955
	Steel: Automotive	28%	10408.33		1	0.28	
	Steel: Mechanical engineering	9%	21645.49		0.7	0.063	
	Energy	6%	14430.32		0.7	0.042	
	Electric appliances	5%	3882.91		1	0.05	
	Others	8%	4425.10		1	0.08	

**Lead（Pb）**	Batteries	93%	34570.52	44892.01	1	0.93	0.973
	Lead compounds	4%	3106.33		0.7	0.028	
	Solders	3%	7215.16		0.5	0.015	
**Lithium（Li）**	Batteries	51%	18958.03	54552.16	0.9	0.459	0.768
	Ceramics and glass	13%	10095.57		0.7	0.091	
	Lubricating grease	15%	11648.73		0.7	0.105	
	Pharmaceuticals	8%	6212.66		0.7	0.056	
	Dye adsorbents	5%	3882.91		0.5	0.025	
	Catalysts	4%	3106.33		0.5	0.02	
	Others	4%	647.94		0.3	0.012	
**Magnesium（Mg）**	aluminum-based alloys (packaging, transportation, other applications)	29%	22520.88	240505.4	0.7	0.203	0.939
	Magnesium die-casting	34%	81771.84		0.7	0.238	

	Metal reduction	14%	33670.76		0.7	0.098	
	Steel desulphurisation	11%	26455.59		0.5	0.055	
	Nodular cast iron	10%	24050.54		0.7	0.07	
	Others	2%	4810.11		0.7	0.014	
**Manganese（Mn）**	Steel and iron	90%	216454.86	220398.71	1	0.9	1
	Aviation	5%	1858.63		1	0.05	
	Batteries	3%	792.02		1	0.03	
	Agriculture	2%	1293.2		1	0.02	
**Carbon（C）**	Electrodes	39%	14497.31	84562.19	1	0.39	0.749
	Others	28%	4535.58		0.5	0.14	
	Refractories	20%	48101.08		0.7	0.14	
	Lubricants	6%	4659.49		0.5	0.03	
	Foundries	5%	12025.27		0.7	0.035	

	Brake pads	2%	743.45		0.7	0.014	
**Nickel（Ni）**	Stainless steel	82%	197214.43	218998.89	0.7	0.574	0.713
	Batteries	9%	2376.05		0.7	0.063	
	Electroplating	3%	7215.16		0.7	0.021	
	Alloy steel	5%	12025.27		1	0.05	
	Others	1%	167.97		0.5	0.005	
**Phosphorus（P）**	Food additives	10%	6466	131486.08	1	0.1	0.98
	Fertilizers, detergents	43%	33393.03		1	0.43	
	Food&medicine	37%	88987.00		1	0.37	
	Electronic parts	10%	2640.06		0.8	0.08	
**Platinum Group Metals（PGMs ）**	Catalysts	55%	132277.97	152999.50	1	0.55	0.864
	Jewellery	17%	1130.13		0.5	0.085	
	Electronics	10%	2640.06		1	0.1	

	Chemical & Electrochemical	7%	5436.07		1	0.07	
	Others	6%	3879.60		0.5	0.03	
	Medical alloys	3%	7215.16		0.3	0.009	
	Petroleum Production	2%	420.51		1	0.02	
**Rare Earth （REEs）**	Catalysts	19%	14755.06	70998.07	1	0.19	0.954
	Magnets	25%	6600.15		1	0.25	
	Polishing powder	14%	10872.15		1	0.14	
	Batteries (NiMH)	14%	5204.16		1	0.14	
	Metallurgy	9%	21645.49		0.7	0.063	
	Automotive catalyst	7%	2602.08		0.9	0.063	
	Glass	6%	4659.49		0.9	0.054	
	Phosphor	6%	4659.49		0.9	0.054	
**Silicon （Si）**	Organosilicon	44%	34169.61	117467.48	0.7	0.308	0.796

	Silicon alloy	32%	76961.73		1	0.32	
	Polysilicon	24%	6336.14		0.7	0.168	
**Silver (Ag)**	Jewellery, Silverware, Coins and Medals	38%	29510.12	62280.92	0.7	0.266	0.72
	Electronics	30%	7920.18		1	0.3	
	Others	29%	22520.88		0.5	0.145	
	Photography	3%	2329.75		0.3	0.009	
**Sulphur (S)**	Chemical applications	90%	69892.38	74346.11	1	0.9	0.967
	Pharmacy	3%	570.82		0.9	0.027	
	Rubber products	5%	3882.91		0.8	0.04	
**Tellurium (Te)**	Metallurgy	42%	11088.25	82067.85	0.3	0.126	0.406
	Chemical industries and catalysts	21%	5544.13		0.7	0.147	
	Photovoltaics	26%	62531.40		0.3	0.078	
	Thermoelectrics, others	11%	2904.07		0.5	0.055	

**Titanium（Ti）**	Chemical industries	46%	35722.77	104123.44	0.3	0.138	0.456
	Aviation	15%	5575.89		0.5	0.075	
	Sports & Recreation	15%	36075.81		0.5	0.075	
	Metallurgy	8%	19240.43		0.7	0.056	
	Salt manufacturing	8%	6212.66		0.7	0.056	
	Others	8%	1295.88		0.7	0.056	
**Vanadium（V）**	Iron vanadium and nitrogen vanadium alloy Iron vanadium and nitrogen vanadium alloy	85%	204429.59	210005.48	0.7	0.595	0.69
	Aircraft engines, blades	8%	2973.81		0.7	0.056	
	Magnets	5%	1858.63		0.5	0.025	
	superconducting alloy	2%	743.45		0.7	0.014	
**Zinc（Zn）**	Galvanizing	48%	115442.59	143067.91	0.7	0.336	0.679
	Die casting	18%	6691.07		0.5	0.09	

	Chemicals	9%	6989.24		0.7	0.063	
	Brass and Bronze	13%	10095.57		1	0.13	
	Batteries	9%	3345.53		0.5	0.045	
	Others	3%	503.92		0.5	0.015	
**Antimony （Sb）**	Flame Retardants	60%	46594.92	57269.14	0.7	0.42	0.62
	Lead-acid batteries (automotive) &Bearings	20%	7434.52		0.7	0.14	
	Pharmaceuticals &Others	20%	3239.7		0.3	0.06	
**Germanium （Ge）**	Fibre optic	30%	7920.18	68767.84	1	0.3	0.81
	Infrared optic	20%	15531.64		0.7	0.14	
	Catalysts (polymers)	20%	5280.12		0.8	0.16	
	Parts for electrical and solar equipment	15%	3960.09		0.7	0.105	
	Others	15%	36075.81		0.7	0.105	
**Cadmium**	Batteries (Ni-Cd)	86%	22704.52	33064.09	0.5	0.43	0.518

**（Cd）**	Pigments	9%	6989.24		0.7	0.063	
	Paints	4%	3106.33		0.5	0.02	
	Alloys, solar panels	1%	264.01		0.5	0.005	
**Arsenic（As）**	Chemicals	26%	6864.16	33584.08	0.5	0.13	0.648
	LED	25%	6600.15		0.7	0.175	
	Alloys	17%	13201.89		0.7	0.119	
	Solar	17%	4488.10		0.7	0.119	
	Others	15%	2429.78		0.7	0.105	
Molybdenum（Mo）	Iron and Steel Industry	80%	192404.32	204981.59	1	0.8	1
	Chemicals	10%	7765.82		1	0.1	
	Electronics	4%	671.89		1	0.04	
	Pharmaceuticals	2%	1553.164		1	0.02	
	Agriculture	4%	2586.40		1	0.04	

Tungsten（W）	Hard metal	51%	122657.75	202900.94	0.7	0.357	0.7
	Tungsten steel	30%	72151.62		0.7	0.21	
	Tungsten material	13%	3432.08		0.7	0.091	
	Chemicals	6%	4659.49		0.7	0.042	
Gold（Au）	Jewellery	50%	38829.10	49939.07	0.7	0.35	0.785
	Electronics	37%	9768.22		1	0.37	
	Coins	8%	531.82		0.5	0.04	
	Others	5%	809.93		0.5	0.025	
Bismuth (Bi)	Kinescope	40%	10560.24	77548.71	1	0.4	0.918
	Pharmaceuticals	28%	21744.30		0.9	0.252	
	Alloys and solder	13%	31265.70		0.8	0.104	
	Metallurgical additive	18%	13978.48		0.9	0.162	
Beryllium	Engineering& aerospace	22%	52911.19	94130.89	0.9	0.198	0.83

（Be）	Electronics	21%	5544.13		0.9	0.189	
	Automotive electronics	16%	5947.62		0.7	0.112	
	Military industry	9%	21645.49		0.9	0.081	
	Telecommunications	8%	2112.05		1	0.08	
	Energy	7%	2602.08		0.7	0.049	
	Pharmaceuticals	1%	776.58		0.9	0.009	
	Others	16%	2591.76		0.7	0.112	
Boron（B）	Glass&Ceramics	80%	62126.56	70895.16	0.5	0.4	0.5
	Insecticides	8%	6212.66		0.5	0.04	
	Semiconductor	6%	1584.04		0.5	0.03	
	Others	6%	971.91		0.5	0.03	
Cesium（Cs）	Optical instruments	32%	9573.86	33173.30	1	0.32	0.992
	Solar batteries	20%	7434.52		1	0.2	

	Monitors in Navigation satellite	40%	14869.04		1	0.4	
	Others	8%	1295.88		0.9	0.072	
Mercury（Hg）	Catalysts	70%	11758.04	25364.60	0.9	0.63	0.805
	Scientific measuring instrument	9%	2692.65		0.5	0.045	
	Pharmaceuticals	8%	6212.66		0.5	0.04	
	Vapor lamp	5%	3882.91		0.5	0.025	
	Electrodes	3%	485.96		0.5	0.015	
	Fulminating mercury	5%	332.39		1	0.05	
Niobium（Nb）	Steel	85%	204429.59	240505.40	0.7	0.595	0.7
	Superalloy	15%	36075.81		0.7	0.105	
铼（Re）	Superalloy in turbine engines	80%	29738.08	36774.82	0.9	0.72	0.905
	Petroleum reforming catalyst	15%	3153.83		1	0.15	
	Heating devices	5%	3882.91		0.7	0.035	

Rhenium （Rb）	Military industry	33%	79366.78	97952.02	0.7	0.231	0.693
	Science and technology	33%	9873.04		0.7	0.231	
	Photocell	33%	8712.20		0.7	0.231	
Scandium （Sc）	Aluminium base alloy	58%	139493.13	159807.75	0.8	0.464	0.83
	Laser	9%	2376.05		1	0.09	
	Semiconductor	15%	3960.09		1	0.15	
	Electric light material	18%	13978.48		0.7	0.126	
Selenium （Se）	Metallurgy	10%	24050.54	136001.2	1	0.1	0.71
	Glass	35%	27180.37		0.7	0.245	
	Agriculture	5%	3233.00		0.7	0.035	
	Chemicals and pigments	10%	7765.82		0.5	0.05	
	Electronics	30%	72151.62		0.7	0.21	
	Others	10%	1619.85		0.7	0.07	

Strontium（Sr）	Drilling fluid	64%	13456.32	49340.31	0.7	0.448	0.79
	Ceramic ferrite magnet	12%	9318.98		1	0.12	
	Pyrotechnic signal	12%	9318.98		1	0.12	
	Electrolytic zinc	3%	7215.16		0.7	0.021	
	Alloys	3%	7215.16		1	0.03	
	Pigments	3%	2329.75		1	0.03	
	Others	3%	485.96		0.7	0.021	
Tantalum（Ta）	Aerospace alloys	18%	6691.07	81863.47	1	0.18	0.754
	Automotive	25%	60126.35		0.7	0.175	
	Electronics	43%	11352.26		0.7	0.301	
	Instruments and apparatus	8%	2721.89		0.7	0.056	
	Others	6%	971.91		0.7	0.042	
Tin（Sn）	Electronic welding	70%	168353.78	198533.05	0.9	0.63	0.816

	Plating	8%	19240.43		0.7	0.056	
	Chemicals	12%	9318.98		0.5	0.06	
	Others	10%	1619.85		0.7	0.07	
Zirconium（Zr）	Ceramics	53%	41158.85	97383.82	0.7	0.371	0.73
	Casting	14%	33670.76		0.7	0.098	
	Emulsion	5%	3882.91		0.7	0.035	
	Fireproofing	10%	7765.82		1	0.1	
	Chemicals	13%	10095.57		0.7	0.091	
	Others	5%	809.93		0.7	0.035	

Table S2 Main industries of national economy and total economic value

National industries classification	Value (V_i)
Agriculture, forestry, animal husbandry and fishery	64660
Mining industry	21025.5
Manufacturing industry	240505.4
Electricity and heat power	16797.2
Building industry	55313.8
Chemical consumption	77658.2
Transportation, warehousing and postal services	37172.6
Electronics and information transmission	26400.6
Financial industry	65395
Realty industry	53965.2
Leasing and business service	21887.8
Scientific research and technical services	16198.5
Water, environmental and public utility management industries	4762.8
Residential services, repairs and other services	14704.4
Education	29918.3
Health and social work	19027.3
Culture, sports and entertainment	6647.8
Public administration, social security and social organizations	34023.6
Gross National Product（GDP）	820754.3

The domestic values of different industries are given in billion CYN. The national industries classification and their corresponding values are from China Statistical Yearbook.

Table S3 Results of domestic economy index and characteristics value parameter of resources

**Materials**	Characteristics value parameter **(Q_M)**	Domestic economy index **(DE_M)**
PGMs	6.09	11.21
V	3.68	9.32
Nb	2.99	8.66
Sc	4.37	8.4
Rb	6.52	7.7
Co	2.69	7.13
Mo	2.69	6.66
W	2.63	6.43
Ni	2.27	5.91
Sn	2.41	5.76
Cr	1.94	5.43
Be	4.03	4.55
REEs	5.26	4.47
Mg	1.43	4.13
Se	2.37	3.89
Ta	3.82	3.79
B	4.2	3.57
Au	5.87	3.52
Cu	1.95	3.51
Ge	4.11	3.41
Mn	1.28	3.39
Zr	2.67	3.13
Ag	3.9	2.92
Te	2.87	2.84
Zn	1.49	2.57
Ga	3.26	2.54
Ti	2.03	2.54
Cs	5.97	2.39
Re	4.67	2.05
In	3.23	1.94
Si	1.3	1.82

Li	2.66	1.75
Bi	1.8	1.68
Al	1.41	1.41
Sb	1.81	1.25
P	0.77	1.24
Sr	1.83	1.08
Hg	3.05	0.91
C	0.89	0.89
Fe	0.83	0.79
Pb	1.43	0.77
Cd	1.42	0.57
As	1.18	0.47
S	0.01	0.01

Table S4 The recycling rate and import reliance of resources

**Materials**	**Recycling rate (ρ_M)**	**Domestic consumption (*Do*+*Im*-*Ex*)**	**Domestic production (*Do*)**	**Import reliance (TR_M)**
Li	14	3.24	0.75	4.3
Be	23	0.00794	0.007	1.1
B	0	56	16	3.5
C	0	61.4	70	0.9
Mg	33	45	90	0.5
Al	45	3470	3500	1.0
Si	0	37.4	33	1.1
P	0	7912	11000	0.7
S	0	4578	3525	1.3
Sc	0	0.00011	0.000179	0.6
Ti	91	7.86	7.5	1.0
V	0	7.2	6	1.2
Cr	28	10	2	5.0
Mn	53	330	130	2.5
Fe	90	83500	80000	1.0
Co	29	2	0.2	10.0
Ni	47	111.1	11	10.1
Cu	35	1248.2	895	1.4
Zn	25	680	568	1.2
Ga	0	0.017	0.031	0.5

Ge	30	0.0075	0.00085	8.8
As	0	4.74	1.8	2.6
Se	0	0.28	0.04	7.0
Rb	0	0.00042	0.0004	1.1
Sr	0	7.8	5	1.6
Zr	0	112	14	8.0
Nb	20	1.5	0.075	10.0
Mo	30	9.3	6.3	1.5
Ag	17	0.82	0.36	2.3
Cd	15	0.76	0.82	0.9
In	0	0.017	0.03	0.6
Sn	24	17.4	14	1.2
Sb	0	7	10	0.7
Te	0	0.015	0.03	0.5
Cs	85	NA	0.0001	7.0
Ta	30	0.055	0.009	6.1
W	25	4.7	6.5	0.7
Re	50	NA	0.00025	2.0
Au	87	0.12	0.04	3.0
Hg	5	0.1	0.144	0.7
Pb	73	520	210	2.5
Bi	5	0.76	1.4	0.5
REEs	0	6500	5500	1.2
PGMs	60	40	3.95	10.1

Table S5 World government index for countries

**Country**	**WGI**	**WGI-scaled**	**Country**	**WGI**	**WGI-scaled**
China	-0.31	2.69	Philippines	-0.34	2.66
Canada	1.59	4.59	Indonesia	-0.14	2.86
Australia	1.58	4.58	Zambia	-0.36	2.64
Russia	-0.64	2.36	Ukrain	-0.68	2.32
India	-0.11	2.89	Ghana	0.05	3.05
US	1.24	4.24	Uzbekistan	-0.95	2.05
norway	1.76	4.76	France	1.12	4.12
arab emirates	0.66	3.66	Sweden	1.7	4.7
bahrain	-0.23	2.77	portugal	1.07	4.07
iceland	1.55	4.55	Israel	0.66	3.66
bolivia	-0.55	2.45	Cote d'lvoire	-0.5	2.5
burma	0.94	3.94	Gabon	-0.74	2.26
tajikistan	-1.21	1.79	Georgia	0.43	3.43
turkey	-0.48	2.52	Malaysia	0.47	3.47

belgium	1.18	4.18	Kyrgyastan	-0.63	2.37
iran	-1	2	Armenia	-0.13	2.87
morocco	-3	0	Mongolia	0.02	3.02
namibia	0.3	3.3	Thailand	-0.28	2.72
brazil	-0.24	2.76	Vietnam	-0.35	2.65
japan	1.34	4.34	Poland	0.65	3.65
kazakhstan	-0.32	2.68	Bhutan	0.56	3.56
korea,republic	0.91	3.91	Spain	0.81	3.81
laos	-0.77	2.23	Italy	0.49	3.49
mexico	-0.35	2.65	Kuwait	-0.11	2.89
argentina	0.007	3.007	Qatar	0.35	3.35
chile	1.006	4.006	Saudi Arabia	-0.23	2.77
germany	1.5	4.5	Venezuela	-1.75	1.25
peru	-0.13	2.87	Burundi	-1.43	1.57
Netherlands	1.69	4.69	Ethiopia	-0.83	2.17
Finland	1.76	4.76	Nigerial	-0.74	2.26
South africa	0.13	3.13	Rwanda	0	3
Zimbabwe	-1.19	1.81	Bulgaria	0.24	3.24
Congo(Kinshasa)	-1.11	1.89	Kenya	-0.57	2.43
Cuba	-0.46	2.54	mozambique	-0.78	2.22
Madagascar	-0.75	2.25	Senegal	-0.08	2.92
New Caledonia	-0.77	2.23	pakistan	-0.97	2.03
Papua New Guinea	-0.58	2.42	jordan	-0.08	2.92

Table S6 Chinese production share and Herfindahl-Hirschman index of resources

Materials	Chinese production share（%）	HHI
Li	9.74	1.63
Be	26.92	2
B	7.08	1.35
C	0.64	1.15
Mg	81.82	1.82
Al	56.25	0.89
Si	64.29	1.16
P	45.83	0.62
S	22.03	0.28
Sc	50	1.15
Ti	40	0.85
V	54.79	1.01

Cr	0	0.7
Mn	6.84	0.48
Fe	14.67	0.86
Co	1.43	0.99
Ni	4.07	0.4
Cu	8	0.42
Zn	33.08	0.42
Ga	96.88	2.53
Ge	65.38	1.16
As	72.73	1.43
Se	33.21	0.72
Rb	40	1.05
Sr	22.73	0.92
Zr	5.71	0.97
Nb	0	1.5
Mo	44.83	0.81
Ag	13.33	0.32
Cd	32.8	0.52
In	39.47	0.9
Sn	27.42	0.55
Sb	62.5	1.16
Te	61.7	1.15
Cs	0	1.57
Ta	5.56	0.54
W	82.35	1.84
Re	5.1	1.48
Au	12.73	0.17
Hg	87.5	2.07
Pb	46.67	0.68
Bi	73.68	1.53
REEs	62.86	1.22
PGMs	0	1.68

Table S7 Toxicity degree value of metals

**Materials**	**T_M**
Li	0.1
Be	1
B	0.1
Mg	0.1
Al	0.67
Sc	0.67
Ti	0.67
V	0.67
Cr	1
Mn	0.67
Fe	0.67
Co	0.67
Ni	0.67
Cu	0.67
Zn	0.67
Ga	0.67
Ge	0.67
As	1
Se	0.1
Rb	0.1
Sr	0.1
Zr	0.1
Nb	0.34
Mo	0.34
Ag	0.67
Cd	1
In	1
Sn	1
Sb	0.67
Te	0.67
Cs	0.34
Ta	0.34
W	0.67

Re	0.34
Au	0.67
Hg	1
Pb	1
Bi	0.67
REEs	0.34
PGMs	0.1

Table S8 Annual amount of waste gas, waste water and waste solid emission in China and Environmental Performance Index for China

Q_{wg}	Q_{wl}	Q_{ws}	EPI_{CN}
2930.49	6996610	338528.9	50.74
